# Endoscopic vs. microscopic transsphenoidal surgery for Cushing’s disease: a systematic review and meta-analysis

**DOI:** 10.1007/s11102-018-0893-3

**Published:** 2018-05-16

**Authors:** Leonie H. A. Broersen, Nienke R. Biermasz, Wouter R. van Furth, Friso de Vries, Marco J. T. Verstegen, Olaf M. Dekkers, Alberto M. Pereira

**Affiliations:** 1 Department of Medicine, Division of Endocrinology, Leiden University Medical Centre, Albinusdreef 2, 2333 ZA Leiden, The Netherlands; 2 Center for Endocrine Tumors Leiden (CETL), Leiden University Medical Center, Albinusdreef 2, 2333 ZA Leiden, The Netherlands; 3 Department of Neurosurgery, Leiden University Medical Centre, Albinusdreef 2, 2333 ZA Leiden, The Netherlands; 4 Department of Clinical Epidemiology, Leiden University Medical Center, Albinusdreef 2, 2333 ZA Leiden, The Netherlands

**Keywords:** Cushing’s disease, Transsphenoidal surgery, Endoscopic surgery, Microscopic surgery

## Abstract

**Purpose:**

Systematic review and meta-analysis comparing endoscopic and microscopic transsphenoidal surgery for Cushing’s disease regarding surgical outcomes (remission, recurrence, and mortality) and complication rates. To stratify the results by tumor size.

**Methods:**

Nine electronic databases were searched in February 2017 to identify potentially relevant articles. Cohort studies assessing surgical outcomes or complication rates after endoscopic or microscopic transsphenoidal surgery for Cushing’s disease were eligible. Pooled proportions were reported including 95% confidence intervals.

**Results:**

We included 97 articles with 6695 patients in total (5711 microscopically and 984 endoscopically operated). Overall, remission was achieved in 5177 patients (80%), with no clear difference between both techniques. Recurrence was around 10% and short term mortality < 0.5% for both techniques. Cerebrospinal fluid leak occurred more often in endoscopic surgery (12.9 vs. 4.0%), whereas transient diabetes insipidus occurred less often (11.3 vs. 21.7%). For microadenomas, results were comparable between both techniques. For macroadenomas, the percentage of patients in remission was higher after endoscopic surgery (76.3 vs. 59.9%), and the percentage recurrence lower after endoscopic surgery (1.5 vs. 17.0%).

**Conclusions:**

Endoscopic surgery for patients with Cushing’s disease reaches comparable results for microadenomas, and probably better results for macroadenomas than microscopic surgery. This is present despite the presumed learning curve of the newer endoscopic technique, although confounding cannot be excluded. Based on this study, endoscopic surgery may thus be considered the current standard of care. Microscopic surgery can be used based on neurosurgeon’s preference. Endocrinologists and neurosurgeons in pituitary centers performing the microscopic technique should at least consider referring Cushing’s disease patients with a macroadenoma.

**Electronic supplementary material:**

The online version of this article (10.1007/s11102-018-0893-3) contains supplementary material, which is available to authorized users.

## Introduction

Cushing’s disease is caused by an adrenocorticotropic hormone (ACTH)-secreting pituitary adenoma, with an estimated incidence of 1.2–2.4 per million each year [1]. The resulting excess of glucocorticoids induces insulin resistance, dyslipidemia, central obesity, hypercoagulability, and increases the risk of osteoporosis, hypertension, and neuropsychiatric disorders [2, 3]. First-choice treatment for Cushing’s disease is transsphenoidal pituitary surgery, with selective adenoma removal [4]. Despite biochemical cure, mortality risk in Cushing’s disease patients remains increased [5].

Two main techniques have been used for transsphenoidal pituitary surgery: microscopic and endoscopic surgery. Furthermore, the microscopic and endoscopic techniques have been used in combination, in which the endoscope was used to visually confirm findings of the microscope. The microscopic technique was the established method to perform transsphenoidal surgery, until the first reports on endoscopic pituitary surgery were published, starting in 1992 [6]. With the operating microscope, intraoperative differentiation of pathologic tissue from normal tissue is achieved by providing three-dimensional vision in a direct line to the pituitary [7, 8]. Endoscopic pituitary surgery provides a broader field of vision using endoscopes with various angles in close proximity to the pituitary, however losing the three-dimensional vision and thus depth perception [6, 8]. From the introduction of the endoscope in transsphenoidal surgery, most surgical centers have chosen for microscopy or endoscopy. Only few small cohort studies have compared the microscopic and endoscopic surgical techniques in Cushing’s disease performed in the same center [9–12]. No clear differences in remission rate or surgical morbidity between microscopic and endoscopic surgery could be shown. However, the studies had only limited statistical power [9].

Several systematic reviews have compared endoscopic and microscopic surgical techniques in a heterogeneous population of patients with various pituitary adenomas. These studies have found a reduced rate of some complications (postoperative diabetes insipidus, rhinological complications), but an increased rate of other complications (vascular complications, cerebrospinal fluid leak, anterior pituitary hormone deficiency) for the endoscopic technique [8, 13, 14]. These differences in outcomes may partially be explained by the surgeon’s attempt for a more radical tumor excision with the newer endoscopic technique with better vision, by the larger proportion of more challenging macroadenomas and re-operations reported in literature, and by improved rhinological care by an otolaryngologist after endoscopic surgery [8, 13, 14]. Until now, no systematic review has been published comparing the microscopic to the endoscopic surgical technique in Cushing’s disease. Convincing evidence supporting the choice for one of both techniques in the treatment of Cushing’s disease, either based on treatment results or complication rate, is thus lacking.

### Study aims

The primary aims of this systematic review were to compare remission and recurrence rate, and mortality, after microscopic vs. endoscopic transsphenoidal pituitary surgery for Cushing’s disease. Secondary study aims were to compare complication rates, remission and recurrence rates stratified by tumor size, and percentage remission after a repeat transsphenoidal surgical procedure.

## Methods

### Eligibility criteria

Randomized controlled trials and cohort studies in Cushing’s disease assessing outcomes after endoscopic or microscopic transsphenoidal surgery were eligible. Studies describing endoscope-assisted microscopic surgery were considered microscopic surgery. Single-arm studies as well as direct comparisons were considered, mainly because we did not expect many direct comparisons in a single cohort. Study outcomes of interest were remission rate, recurrence rate, short and long term mortality risk, and complications of surgery. Studies reporting outcomes after primary as well as after repeat transsphenoidal surgery were eligible. Studies reporting < 10 patients with Cushing’s disease per treatment group were excluded to minimize the risk of selection bias. Articles were also excluded if the study included children only, if the study did not clearly report which surgery type was performed, or if no distinction between surgery types was made in the analysis. Articles including patients with selective adenomectomy as well as partial or total hypophysectomy were included as long as total hypophysectomy did not exceed 5%. If described separately, patients with total hypophysectomy were excluded from analyses. If multiple articles described (partially) the same population, the article with the largest cohort was included per analysis. Articles irretrievable online were requested by contacting the authors. Articles still irretrievable, but with sufficient data mentioned in the abstract for reliable eligibility assessment and data extraction, were included. Only articles in English were considered.

### Search strategy

To identify potentially relevant articles, PubMed, Embase, Web of Science, COCHRANE Library, CENTRAL, Emcare, LWW, ScienceDirect and Wiley were systematically searched in February 2017 in cooperation with a specialized librarian (see Online Resource 1 for the complete search strategy). References of included articles were searched and the search strategy was manually extended in PubMed with the search term ‘pituitary adenoma’ to find more potentially eligible studies.

### Data extraction

All identified articles were imported in endnote 8 (Thomson Reuters, Philadelphia, PA, USA). Studies were screened by title and abstract and potentially relevant articles were reviewed in detail to assess eligibility. Potentially relevant articles were screened and reviewed by two reviewers independently and disagreement was solved by consensus. The meta-analysis of observational studies in epidemiology (MOOSE) guidelines were used for reporting [15].

### Risk of bias assessment

For risk of bias analysis we used a component approach. Risk of bias was assessed by two independent reviewers for all included studies using the following components, which could potentially bias a reported association between surgical technique and outcome:

1. Inclusion of patients (consecutive inclusion or a random sample is considered low risk of bias)
2. Loss to follow-up (< 5% is considered low risk of bias)
3. Criteria for diagnosis of Cushing’s disease (see below)
4. Clear reporting of criteria for main study outcome. For most studies the main outcome is remission of Cushing’s disease. If remission is not a study outcome, studies will be checked for reporting criteria for their primary study outcome, most often one or more complications of treatment.

As criteria for diagnosis of Cushing’s disease vary widely over time and per study center, and study outcomes also vary per included article, mentioning the criteria for diagnosis and study outcome is considered a low risk of bias. Classification of interventions is not considered in this risk of bias analysis, because the interventions of interest are one-time procedures and therefore unlikely to be misclassified.

Risk of bias analysis was used to explore potential heterogeneity. As most studies did not compare the two surgical techniques directly, confounding was not judged at the study level, but was assessed by comparing baseline characteristics between microscopically and endoscopically treated patients. Variables influencing the choice of treatment as well as co-interventions that could affect treatment outcome are reported.

### Study endpoints

The main outcomes of this study were the percentage of patients reaching remission, the recurrence rate, and the short term mortality risk after microscopic and endoscopic transsphenoidal pituitary surgery for Cushing’s disease. Secondary outcomes were complication rates, rates of remission and recurrence stratified by tumor size, and the percentage of patients to reach remission after a repeat transsphenoidal surgical procedure. Because of the low number of studies with direct comparisons, percentages were reported per surgical technique.

Remission was considered direct postoperatively (until 6 months post-surgery). Hydrocortisone dependency was calculated as a percentage of the total patient population to maintain comparability with remission rate. Disease recurrence was estimated as percentage of the patients with initial remission. Mortality risk was analysed for short-term mortality (< 3 months after surgery). Long-term mortality risk (≥ 3 months after surgery) was not analysed, as time since surgery was often unclear. Articles reporting mortality without mentioning time since surgery were excluded from mortality analyses.

The following complications were assessed: cerebrospinal fluid leak (CSF leak), meningitis, syndrome of inappropriate antidiuretic hormone secretion (SIADH), anterior pituitary hormone deficiency, thromboembolism, bleeding, transient diabetes insipidus, permanent diabetes insipidus, and psychopathology. If an article described diabetes insipidus without specifying the duration, it was excluded from diabetes insipidus analyses.

### Statistical analysis

Percentages were pooled in a random-effects logistic regression model if there were ≥ 5 articles per analysis. A fixed-effects model was used for analyses with < 5 studies. The Freeman-Tukey arcsine transformation was used to stabilize variances, in order to prevent exclusion of studies with 0 or 100% as outcome. All analyses were performed using Stata 11.2 (Stata Corp., College Station, TX, USA).

Sensitivity analyses were performed to assess the potential effect of high risk of selection bias studies by excluding articles in which inclusion of patients was not consecutive or a random sample and/or loss to follow-up was ≥ 5%. Of note, articles not mentioning method of inclusion or loss to follow-up were not excluded in these sensitivity analyses, as this would not leave sufficient articles for analysis (13 for microscopic surgery and one for endoscopic surgery only). Sensitivity analyses were also performed for studies with a study period starting from the year 2000 or later, to assess the potential cohort effect of calendar year of surgery. Finally, sensitivity analyses were performed for studies reporting specific criteria for diagnosis of Cushing’s disease (pituitary imaging or petrosal sinus sampling, and at least one of the following laboratory measurements or tests: increased morning serum cortisol, increased 24-h urinary free cortisol, increased midnight salivary cortisol, no suppression of cortisol after a low dose dexamethasone test combined with a non-suppressed ACTH) to increase reliability of including only Cushing’s disease patients, as well as for studies using at least a low dose dexamethasone test in the determination of remission status to increase test homogeneity, and for studies assessing remission status 3–6 months postoperatively, as this is a more reliable timeframe to correctly assess remission status than direct postoperatively [16].

## Results

### Study selection

**Fig. 1:**
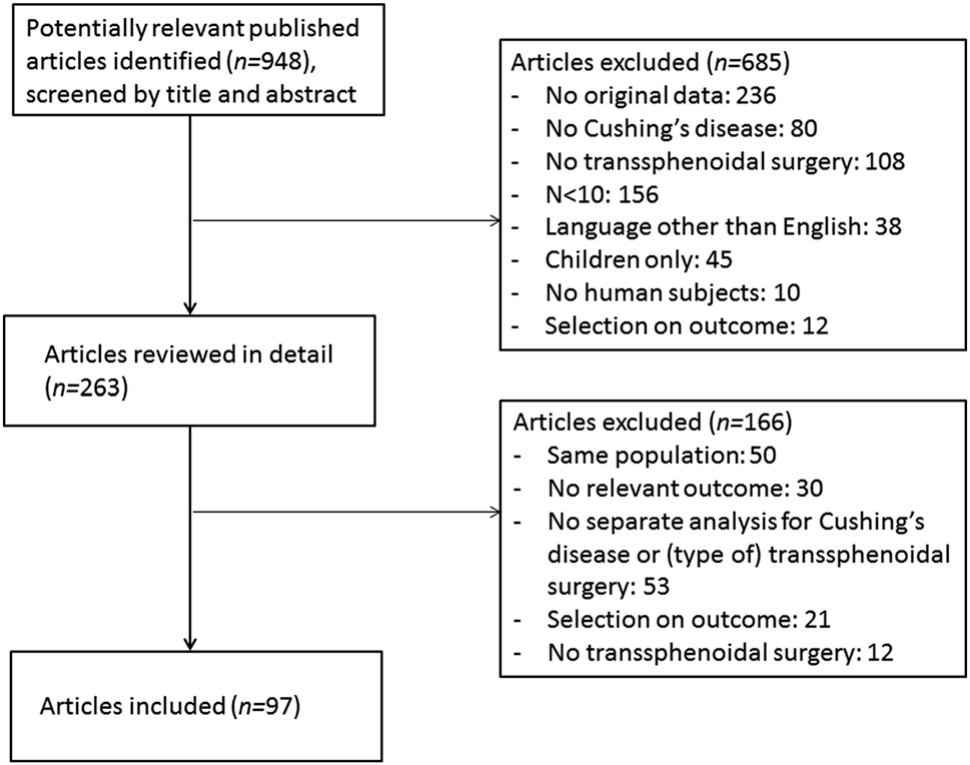
Flow-chart of inclusion of articles in this systematic review

The initial search identified 932 articles. Searching through references of included articles and manually extending the search in PubMed with the search term ‘pituitary adenoma’ identified another 16 articles, thereby yielding a total of 948 articles. After screening the articles by title and abstract, 685 articles were excluded, leaving 263 articles for detailed review. Reasons for exclusion are summarized in Fig. 1. There were 97 articles included in this review, two of which based on abstract only [17, 18].

### Study characteristics (online resource 2)

No RCTs were performed comparing microscopic to endoscopic surgery. There were 71 studies reporting on microscopic surgery only [4, 7, 17, 19–86], 22 studies reporting on endoscopic surgery only [18, 87–107], and four studies from four different centers reporting on both microscopic and endoscopic surgery in the same center [9–12]. Studies reporting on both techniques were entered twice in the tables and analyses, separately for each of the techniques. Articles were published between 1978 and 2017 for microscopic surgery and from 2001 to 2017 for endoscopic surgery. Two included articles reported only results for patients after repeat transsphenoidal surgery [65, 82]. A total of 5711 patients were included for the microscopic technique, and 984 patients for the endoscopic technique.

### Risk of bias assessment

Detailed risk of bias assessment per included article is shown in Online Resource 3. Reported loss to follow-up [reported in 35 studies (36%)] ranged from 0 to 26.9%. Inclusion of consecutive patients or a random sample of patients was explicitly stated in 73 articles (75%). There were 80 articles (82%) that reported the criteria for Cushing’s disease diagnosis, or that referred to the article in which the exact criteria were published. Criteria for main study outcome were reported in 88 articles (91%). Remission of Cushing’s disease was the main study outcome in 83 of these 88 articles (94%).

Differences in baseline characteristics (confounding) are likely as treatment assignment was dependent on calendar year of surgery and center. There were only three articles describing both techniques in the same center and in the same calendar period (see Online Resource 2). Furthermore, there was a slight difference in average age at treatment (microscopy 21.5–50 years; endoscopy 31.9–55.7 years) and in percentage female (microscopy 67–93%; endoscopy 57–95%). Co-interventions that could influence treatment outcome are reported per article in Online Resource 3. Nine included articles (9%) explicitly reported that no co-interventions were used, 20 articles (21%) reported use of co-interventions before or shortly after treatment in part of their included patients. The remaining 68 articles (70%) did not report on co-interventions.

### Study outcomes

For a total of 91 study groups (87 articles), remission was the primary outcome of interest. Overall, remission was obtained in 80% (5177/6484) of patients. There were 48 articles reporting a (short and/or long term) mortality rate, and 60 articles reported the rate of, at least one, complication. For details of study outcomes at the individual study level, see Online Resource 4.

#### Pooled proportions of surgical outcomes: remission, recurrence, mortality, and remission after repeat surgery (Fig. 2; Table 1)

**Fig. 2:**
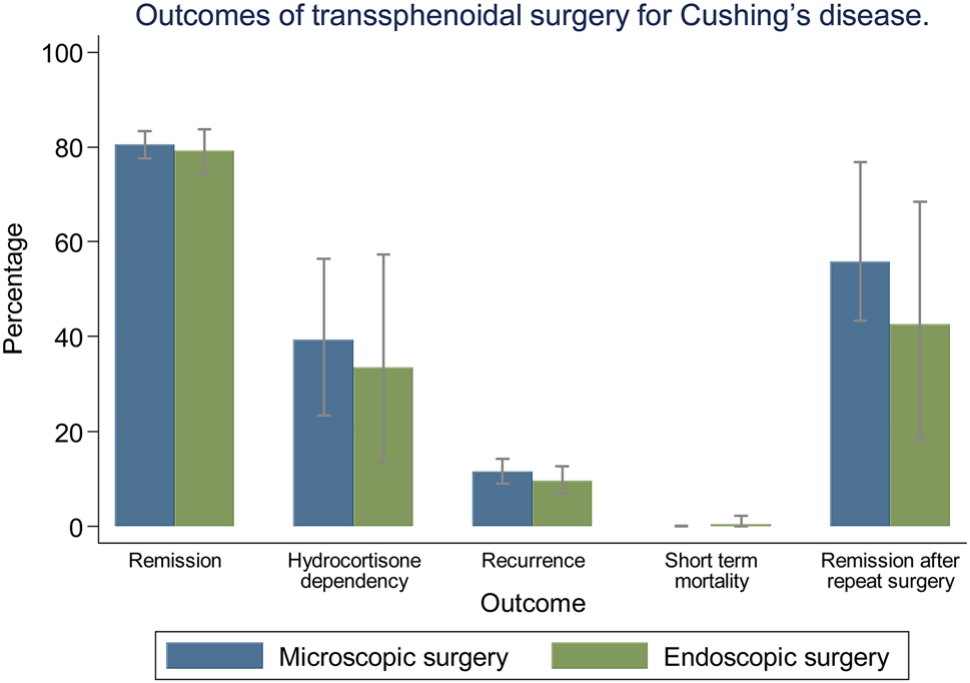
Analysis of surgical outcomes of transsphenoidal surgery for Cushing’s disease. Bars: 95% confidence interval

**Table 1:** Results of meta-analyses comparing microscopic and endoscopic surgery for Cushing’s disease

	Microscopic surgery		Endoscopic surgery	
	Estimated percentage	95% CI	Estimated percentage	95% CI
Meta-analysis of surgical outcomes				
Remission	80.5	77.6–83.3	79.2	74.3–83.8
Hydrocortisone dependency	39.3	23.5–56.4	33.5	13.3–57.3
Recurrence	11.5	9.0–14.3	9.6	6.9–12.7
Short term mortality	0.0	0.0–0.2	0.4	0.0–2.2
Remission after repeat surgery	55.7	43.3–67.8	42.6	18.4–68.4
Meta-analysis of complications				
Cerebrospinal fluid leak	4.0	2.3–6.1	12.9	5.8–22.1
Meningitis	0.6	0.1–1.3	0.1	0.0–1.0
Syndrome of inappropriate antidiuretic hormone secretion	3.5	1.3–6.6	5.2	2.9–8.0
Anterior pituitary hormone deficiency	9.4	5.1–14.8	11.5	5.7–18.8
Thromboembolism	1.2	0.4–2.3	1.5	0.4–3.0
Bleeding	1.9	0.7–3.5	3.7	0.8–8.3
Transient diabetes insipidus	21.7	15.0–29.3	11.3	6.6–17.1
Permanent diabetes insipidus	2.4	1.1–4.1	4.0	2.2–6.3
Psychopathology	0.7	0.0–3.1	–	–
Meta-analysis of surgical outcomes according to tumor size				
Remission for microadenoma	85.5	81.2–89.3	83.9	76.8–90.0
Recurrence for microadenoma	9.8	6.8–13.2	8.1	4.3–12.8
Remission for macroadenoma	59.9	52.0–67.6	76.3	64.3–86.7
Recurrence for macroadenoma	17.0	5.6–31.5	1.5	0.0–6.4

The percentage remission was similar for microscopically and endoscopically treated patients, both reaching around 80% remission. Hydrocortisone dependency was seen in 39.3% (95% confidence interval [CI] 23.5–56.4%) of patients after microscopic surgery and in 33.5% (95% CI 13.3–57.3%) after endoscopic surgery. Recurrence of disease occurred in around 10% of patients after both types of surgery. Average follow-up duration for studies reporting on disease recurrence was 1.0–15.4 years for microscopy and 1.4–5.9 years for endoscopy. Recurrence occurred after an average of 6–76 months in studies using the microscopic technique, and after an average of 24–54 months in studies using the endoscopic technique. Short term mortality was 0.0% (95% CI 0.0–0.2%) for microscopic surgery and 0.4% (95% CI 0.0–2.2%) for endoscopic surgery. The percentage of patients that obtained remission after a repeated transsphenoidal surgical procedure was 55.7% (95% CI 43.3–67.8%) for microscopic surgery, and 42.6% (95% CI 18.4–68.4%) for endoscopic surgery. Measurements of treatment effect were consistent across individual studies, and spread of measurements is reflected by the 95% confidence interval of the outcomes of the analyses.

#### Pooled proportions of complications after transsphenoidal pituitary surgery for Cushing’s disease (Fig. 3; Table 1)

**Fig. 3:**
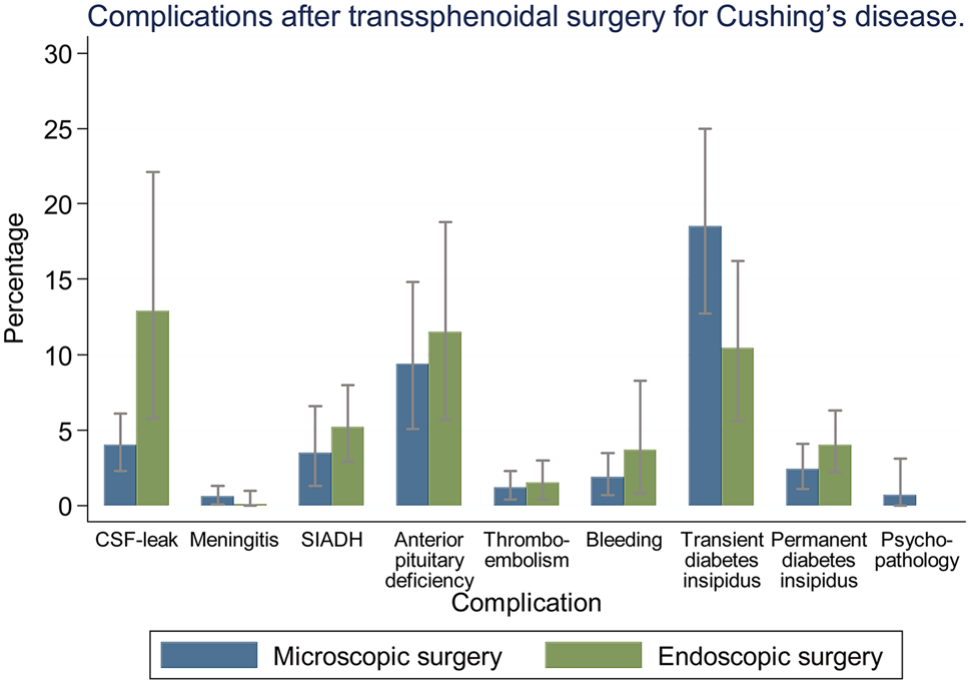
Analysis of complication rates after transsphenoidal surgery for Cushing’s disease. Bars: 95% confidence interval

Cerebrospinal fluid leak was reported less often in patients after microscopic surgery [4.0% (95% CI 2.3–6.1%)], than after endoscopic surgery [12.9% (95% CI 5.8–22.1%)]. Furthermore, SIADH, bleeding and permanent diabetes insipidus were seen slightly less often in patients after microscopic surgery, than in patients after endoscopic surgery. Transient diabetes insipidus was reported more often in patients after microscopic surgery [21.7% (95% CI 15.0–29.3%)], than in patients after endoscopic surgery [11.3% (95% CI 6.6–17.1%)]. Meningitis (around 0.4%), anterior pituitary deficiency (around 10.5%), and thromboembolism (little over 1%), were seen in about equal percentages of patients, regardless of surgical technique. Psychopathology was reported in 0.7% (95% CI 0.0–3.1%) of patients after microscopic surgery. There were no articles on endoscopic surgery reporting on psychopathology.

#### Pooled proportions of remission and disease recurrence according to tumor size (Fig. 4; Table 1)

**Fig. 4:**
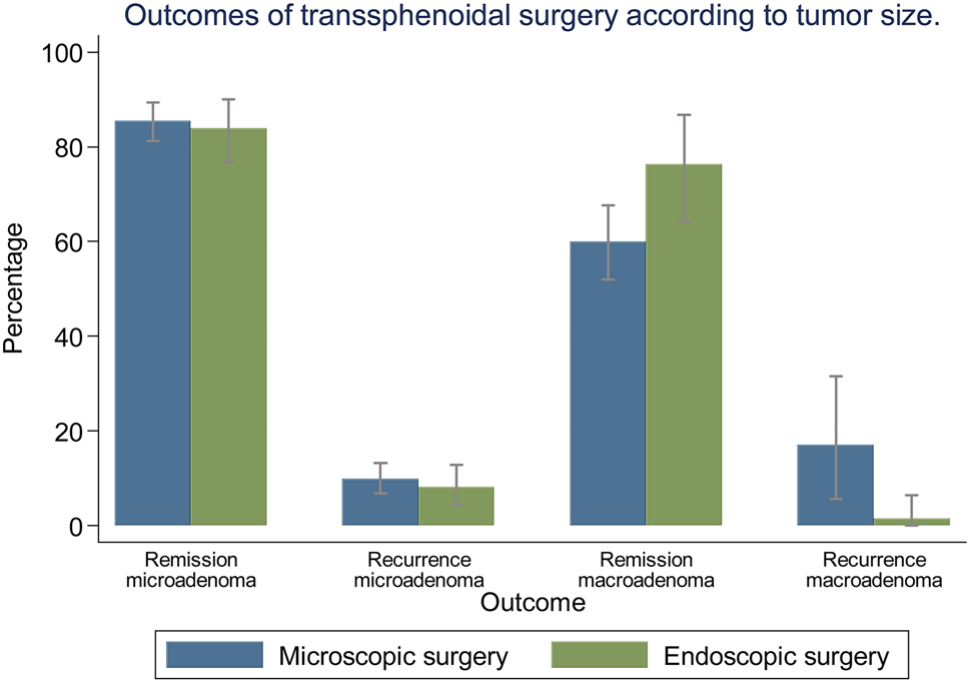
Analysis of surgical outcomes of transsphenoidal surgery for Cushing’s disease, stratified by tumor size. Bars: 95% confidence interval

For microadenomas, the percentage of patients that achieved remission was 85.5% (95% CI 81.2–89.3%) after microscopic surgery vs. 83.9% (95% CI 76.8–90.0%) after endoscopic surgery. Recurrence of disease occurred in 9.8% (95% CI 6.8–13.2%) of patients after microscopic surgery vs. 8.1% (95% CI 4.3–12.8%) after endoscopic surgery.

For macroadenomas, the percentage of patients that achieved remission was 59.9% (95% CI 52.0–67.6%) after microscopic surgery vs. 76.3% (95% CI 64.3–86.7%) after endoscopic surgery. Disease recurrence occurred in 17.0% (95% CI 5.6–31.5%) after microscopic surgery vs. 1.5% (95% CI 0.0–6.4%) after endoscopic surgery.

### Sensitivity analysis

Generally, results from sensitivity analyses were similar to those found in the main analyses. Detailed results for sensitivity analyses and the number of studies per analysis can be found in Online Resource 5.

## Discussion

We performed a systematic review to compare surgical outcomes after microscopic vs. endoscopic transsphenoidal pituitary surgery for Cushing’s disease. Regardless of surgical technique, remission rates were around 80% and recurrence rates around 10% after transsphenoidal surgery. There were no clear differences between surgical techniques regarding mortality, or remission rates after repeat transsphenoidal surgery. Complication rates ranged from 0.1% (for meningitis) to 21.7% (for transient diabetes insipidus), with minor differences between surgical techniques. Remission and recurrence rates for microadenomas were similar for both surgical techniques. However, remission rate was higher for macroadenomas (76.3 vs. 59.9%), with a lower recurrence rate (1.5 vs. 17.0%) after endoscopic surgery than after microscopic surgery. Thus, for macroadenomas only there seems to be an advantage of the endoscopic over the microscopic surgical technique for transsphenoidal treatment of Cushing’s disease.

This is the first systematic review comparing microscopic and endoscopic transsphenoidal pituitary surgery specifically for Cushing’s disease. We found comparable remission and mortality rates for both surgical techniques, which is in line with results of meta-analyses of heterogeneous populations of various pituitary adenomas, and some small cohort studies comparing both techniques directly for Cushing’s disease [8–14]. Differences in complications rates found in meta-analyses of heterogeneous populations of various pituitary adenomas can partially be confirmed by our analysis (reduced rate of transient diabetes insipidus, and increased rate of vascular complications and cerebrospinal fluid leak for the endoscopic technique) [8, 13, 14]. The increased rate of anterior pituitary hormone deficiency for endoscopic transsphenoidal surgery was not found in the present study [14]. The difference in remission rate between the surgical techniques for macroadenomas, but not microadenomas, is in line with the results from a cohort study on multiple pituitary adenomas described separately, that reported an advantage of the endoscopic technique for macroadenomas, but not for microadenomas. This difference was statistically significant for the population as a whole, but was supported by differences in the same direction for all included types of pituitary adenoma, including Cushing’s disease [11].

In interpreting the results, the following study limitations need to be taken into account. Most included studies in this study were single-arm studies, limiting the possibilities of directly comparing microscopic to endoscopic transsphenoidal surgery. However, as treatment assignment in most studies was based on availability of a specific technique in the surgical center, for endoscopy often based on preference of the neurosurgeons after a test period, other baseline characteristics, such as age and gender distribution, are unlikely to have influenced treatment assignment largely. As microscopy was the established surgical technique until the introduction of the endoscope for transsphenoidal surgery for Cushing’s disease, year of surgery varied widely for included studies [108]. For endoscopic surgery, a learning curve has been described [97, 98]. As most studies did not report patient level data, the effect of a potential learning curve per surgical center could not be analyzed in this study. However, to avoid measuring an effect of a collective learning curve, the earliest studies using endoscopic surgery, with study periods starting before the year 2000, were excluded in the previously mentioned sensitivity analysis.

Included studies showed heterogeneity in criteria used for diagnosing Cushing’s disease, both in tests used to determine remission status, and in time period after surgery for assessment of remission status. Sensitivity analyses showed generally comparable results to the main analyses. Differences are likely to have occurred because of the small number of studies included in these sensitivity analyses compared to the number of studies in the corresponding main analyses. However, too many studies did not clearly report loss to follow-up, method of inclusion of patients, or both, preventing us from performing a sensitivity analysis excluding both articles with unclear risk of selection bias as well as high risk of bias, as this restriction would have resulted in one low bias risk article only in endoscopy. Follow-up duration differed between publications, which could potentially lead to a bias in the analysis of recurrences, as this is the only truly long-term outcome. However, given that most recurrences occur early after initial surgery (with only one microscopic study reporting average time to recurrence longer than any average follow-up duration of an endoscopic study), and given that the average follow-up duration for studies reporting on disease recurrence is 1.0–15.4 years for microscopy and 1.4–5.9 years for endoscopy, the bias is probably not very large.

From a pathophysiological perspective, the similar results yielded for microscopic and endoscopic transsphenoidal surgery may be explained by the large percentage of microadenomas in the population of Cushing’s disease patients [109]. For microadenomas, there may not be an advantage in increasing field of vision at the cost of losing three-dimensional vision and thereby depth perception [6, 8]. Most likely, due to their small size, microadenomas are completely within the field of vision regardless of surgical technique. Our results concerning remission and recurrence for microadenomas indeed showed no clear advantage for either technique. For macroadenomas, we did show an advantage of the endoscopic surgical technique. As macroadenomas are larger and more often invasive, a broader field of vision in close proximity to the tumor may aid the neurosurgeon in achieving a complete tumor resection, causing higher remission and lower recurrence rates after endoscopic surgery. In microscopic surgery, these tumors are more often partially out of vision for the neurosurgeon. Unfortunately, due to lack of data, we were unable to perform separate analyses for invasive vs. non-invasive macroadenomas, as well as for small vs. larger microadenomas. The increased rate of cerebrospinal fluid leak after endoscopic transsphenoidal surgery may partially be explained by the neurosurgeon’s attempt to achieve complete tumor resection also in more difficult cases with the newer endoscopic technique, whereas the reduced rate of transient diabetes insipidus may originate from the more precise tumor excision due to improved vision close to the tumor, causing less damage to the posterior lobe of the pituitary. Publication bias has been suggested as partial explanation for the increased rate of cerebrospinal fluid leak after endoscopic surgery, as more often challenging macroadenomas have been described [14].

For most Cushing’s disease patients, this study shows no clear advantage of either microscopic or endoscopic transsphenoidal surgery regarding surgical outcomes and complication rates. For macroadenomas, the endoscopic technique yields better results regarding remission and recurrence rate. These results are present despite the presumed learning curve of the newer endoscopic technique within the study period, although confounding by indication and improved radiological investigations with time cannot be excluded. As most patients with Cushing’s disease have microadenomas [109], there is no reason that all neurosurgical centers treating patients with Cushing’s disease should change to the endoscopic technique. However, there is also no particular reason to keep using the microscopic technique for patients with Cushing’s disease, other than neurosurgeon’s preference. Based on this study, centers that choose to use the microscopic technique should consider referral of patients with Cushing’s disease and a macroadenoma to another surgical center that performs endoscopic surgery.

## Electronic supplementary material

Below is the link to the electronic supplementary material.

Supplementary material 1 (DOCX 23 KB)

Supplementary material 2 (DOCX 33 KB)

Supplementary material 3 (DOCX 45 KB)

Supplementary material 4 (DOCX 43 KB)

Supplementary material 5 (DOCX 26 KB)
