# Supplementary material for: Endoscopic vs. microscopic transsphenoidal surgery for Cushing’s disease: a systematic review and meta-analysis
Source: Pituitary. 2018 May 16;21(5):524–34. doi: 10.1007/s11102-018-0893-3 (PMC6132967; doi:10.1007/s11102-018-0893-3)
Supplement: Supplementary file 1 — Supplementary material 1 (DOCX 23 KB) [file 11102_2018_893_MOESM1_ESM.docx]

**Pituitary
Endoscopic vs. microscopic transsphenoidal surgery for Cushing’s disease: a systematic review and meta-analysis.**

Leonie H.A. Broersen^1,2^, Nienke R. Biermasz^1,2^, Wouter R. van Furth^2,3^, Friso de Vries^1,2^, Marco J.T. Verstegen^2,3^, Olaf M. Dekkers^1,4^, Alberto M. Pereira^1,2^

^1^Department of Medicine, division of Endocrinology, Leiden University Medical Centre, Albinusdreef 2, 2333 ZA, Leiden, The Netherlands
^2^Center for Endocrine Tumors Leiden (CETL), Leiden University Medical Center, Albinusdreef 2, 2333 ZA, Leiden, The Netherlands
^3^Department of Neurosurgery, Leiden University Medical Centre, Leiden, Albinusdreef 2, 2333 ZA, The Netherlands
^4^Department of Clinical Epidemiology, Leiden University Medical Center, Leiden, Albinusdreef 2, 2333 ZA, The Netherlands

Corresponding author: L.H.A. Broersen, [L.H.A.Broersen@lumc.nl](mailto:L.H.A.Broersen@lumc.nl), +31 (0)71-5263082

**Online Resource 1**

Search strategy
**PubMed** (<http://www.ncbi.nlm.nih.gov/pubmed?otool=leiden>)
**(("Cushing Syndrome"[Mesh] OR "Cushings Disease"[tw] OR "Cushings Syndrome"[tw] OR "Cushing's Disease"[tw] OR "Cushing's Syndrome"[tw] OR "Cushing Disease"[tw] OR "Cushing Syndrome"[tw] OR Cushing*[tw] OR "Hypercortisolism"[tw] OR Hypercortisol*[tw] OR "Pituitary ACTH Hypersecretion"[Mesh] OR "Pituitary ACTH Hypersecretion"[tw] OR "Inappropriate ACTH Secretion"[tw]) AND (transsphenoid*[tw] OR trans-sphenoid*[tw] OR "Endoscopy"[Mesh:NoExp]** OR **"Neuroendoscopy"[Mesh] OR Endoscop*[tw]** OR **Neuroendoscop*[tw] OR "Microsurgery"[Mesh:NoExp] OR Microsurg*[tw] OR micro-surg*[tw] OR "microscopic surgery"[tw] OR microscopic surg*[tw]) AND ("Mortality"[mesh] OR "mortality"[Subheading] OR mortalit*[tw] OR "cause of death"[tw] OR "survival rate"[tw] OR "complications"[Subheading:NoExp] OR "Intraoperative Complications"[Mesh] OR "Surgical Injury"[tw] OR "Surgical Injuries"[tw] OR "Surgical Blood Loss"[tw] OR "Intraoperative Awareness"[tw] OR "Malignant Hyperthermia"[tw] OR "Postoperative Complications"[Mesh] OR "complication"[tw] OR "complications"[tw] OR "short term morbidity"[tw] OR "treatment failure"[tw] OR "hydrocortisone dependency"[tw] OR CSF leak*[tw] OR "meningitis"[tw] OR "bleeding"[tw] OR "diabetes insipidus"[tw] OR "SIADH"[tw] OR "anterior pituitary deficiency"[tw] OR "long term morbidity"[tw] OR "recurrent disease"[tw] OR "cardiovascular morbidity"[tw] OR "hypertension"[tw] OR "insulin resistance"[tw] OR "neuropsychiatric morbidity"[tw] OR "Morbidity"[mesh:noexp] OR "morbidiy"[tw] OR "Treatment Failure"[Mesh] OR "Cerebrospinal Fluid Leak"[Mesh] OR "Cerebrospinal Fluid Leak"[tw] OR "Cerebrospinal Fluid Leakage"[tw] OR "Meningitis"[mesh] OR "Hemorrhage" OR hemorrhag*[tw] OR haemorrhag*[tw] OR "Diabetes Insipidus"[Mesh] OR "Inappropriate ADH Syndrome"[Mesh] OR "Inappropriate ADH Syndrome"[tw] OR "anterior pituitary hormone deficiency" OR "Pituitary Hormones, Anterior/deficiency"[mesh] OR "Recurrence"[Mesh] OR "Cardiovascular Diseases/epidemiology"[mesh] OR "Hypertension"[mesh] OR hypertens*[tw] OR "Insulin Resistance"[mesh] OR "metabolic syndrome"[tw] OR neuropsychiat*[tw])) NOT ("Animals"[mesh] NOT "Humans"[mesh]) NOT (("case reports"[ptyp] OR "case report"[ti]) NOT ("Clinical Study"[ptyp] OR "series"[ti] OR "review"[ptyp] OR "review"[ti]))**

**Embase** (<http://ovidsp.ovid.com/ovidweb.cgi?T=JS&PAGE=main&MODE=ovid&D=oemezd>)
**((*"Cushing disease"/ OR *"Cushing syndrome"/ OR "Cushings Disease".ti,ab OR "Cushings Syndrome".ti,ab OR "Cushing's Disease".ti,ab OR "Cushing's Syndrome".ti,ab OR "Cushing Disease".ti,ab OR "Cushing Syndrome".ti,ab OR Cushing*.ti,ab OR "Hypercortisolism".ti,ab OR Hypercortisol*.ti,ab OR "Pituitary ACTH Hypersecretion".ti,ab OR "Inappropriate ACTH Secretion".ti,ab) AND ("transsphenoidal surgery"/ OR transsphenoid*.ti,ab OR trans-sphenoid*.ti,ab OR *"Endoscopy"/** OR ***"Neuroendoscopy"/ OR Endoscop*.ti,ab** OR **Neuroendoscop*.ti,ab OR *"Microsurgery"/ OR Microsurg*.ti,ab OR micro-surg*.ti,ab OR "microscopic surgery".ti,ab OR microscopic surg*.ti,ab) AND (exp "Mortality"/ OR mortalit*.ti,ab OR "cause of death".ti,ab OR "survival rate".ti,ab OR "co".fs OR "Peroperative Complication"/ OR "Surgical Injury".ti,ab OR "Surgical Injuries".ti,ab OR "Surgical Blood Loss".ti,ab OR "Intraoperative Awareness".ti,ab OR "Malignant Hyperthermia".ti,ab OR exp "Postoperative Complication"/ OR "complication".ti,ab OR "complications".ti,ab OR "short term morbidity".ti,ab OR "treatment failure".ti,ab OR "hydrocortisone dependency".ti,ab OR CSF leak*.ti,ab OR "meningitis".ti,ab OR "bleeding".ti,ab OR "diabetes insipidus".ti,ab OR "SIADH".ti,ab OR "anterior pituitary deficiency".ti,ab OR "long term morbidity".ti,ab OR "recurrent disease".ti,ab OR "cardiovascular morbidity".ti,ab OR "hypertension".ti,ab OR "insulin resistance".ti,ab OR "neuropsychiatric morbidity".ti,ab OR "Morbidity"/ OR "morbidiy".ti,ab OR "Treatment Failure"/ OR "liquorrhea"/ OR "Cerebrospinal Fluid Leak".ti,ab OR "Cerebrospinal Fluid Leakage".ti,ab OR exp "Meningitis"/ OR exp "Bleeding" OR hemorrhag*.ti,ab OR haemorrhag*.ti,ab OR exp "Diabetes Insipidus"/ OR "inappropriate vasopressin secretion"/ OR "Inappropriate ADH Syndrome".ti,ab OR "anterior pituitary hormone deficiency".ti,ab OR exp "hormone deficiency"/ OR "Recurrent Disease"/ OR exp "Cardiovascular Disease"/ep OR exp "Hypertension"/ OR hypertens*.ti,ab OR exp "Insulin Resistance"/ OR "metabolic syndrome".ti,ab OR neuropsychiat*.ti,ab)) AND exp "Humans"/ NOT ((exp "case report"/ OR "case report".ti) NOT (exp "Clinical Trial"/ OR "series".ti OR "case study"/ OR exp "review"/ OR "review".ti))**

**Web of Science** (<http://isiknowledge.com/wos>) **(TI=("Cushing disease" OR "Cushing syndrome" OR "Cushings Disease" OR "Cushings Syndrome" OR "Cushing's Disease" OR "Cushing's Syndrome" OR "Cushing Disease" OR "Cushing Syndrome" OR Cushing* OR "Hypercortisolism" OR Hypercortisol* OR "Pituitary ACTH Hypersecretion" OR "Inappropriate ACTH Secretion") AND TS=("transsphenoidal surgery" OR transsphenoid* OR trans-sphenoid* OR "Endoscopy"** OR **"Neuroendoscopy" OR Endoscop*** OR **Neuroendoscop* OR "Microsurgery" OR Microsurg* OR micro-surg* OR "microscopic surgery" OR microscopic surg*)) AND TS=("Mortality" OR mortalit* OR "cause of death" OR "survival rate" OR "Peroperative Complication" OR "Surgical Injury" OR "Surgical Injuries" OR "Surgical Blood Loss" OR "Intraoperative Awareness" OR "Malignant Hyperthermia" OR "Postoperative Complication" OR "complication" OR "complications" OR "short term morbidity" OR "treatment failure" OR "hydrocortisone dependency" OR CSF leak* OR "meningitis" OR "bleeding" OR "diabetes insipidus" OR "SIADH" OR "anterior pituitary deficiency" OR "long term morbidity" OR "recurrent disease" OR "cardiovascular morbidity" OR "hypertension" OR "insulin resistance" OR "neuropsychiatric morbidity" OR "Morbidity" OR "morbidiy" OR "Treatment Failure" OR "liquorrhea" OR "Cerebrospinal Fluid Leak" OR "Cerebrospinal Fluid Leakage" OR "Meningitis" OR "Bleeding" OR hemorrhag* OR haemorrhag* OR "Diabetes Insipidus" OR "inappropriate vasopressin secretion" OR "Inappropriate ADH Syndrome" OR "anterior pituitary hormone deficiency" OR "hormone deficiency" OR "Recurrent Disease" OR "Hypertension" OR hypertens* OR "Insulin Resistance" OR "metabolic syndrome" OR neuropsychiat*) NOT TI=("case report" NOT ("Trial" OR "series" OR "review")) NOT ti=(veterinary OR rabbit OR rabbits OR animal OR animals OR mouse OR mice OR rodent OR rodents OR rat OR rats OR pig OR pigs OR porcine OR horse* OR equine OR cow OR cows OR bovine OR goat OR goats OR sheep OR ovine OR canine OR dog OR dogs OR feline OR cat OR cats)**

**Cochrane** (<http://www.cochranelibrary.com/>)
**(("Cushing disease" OR "Cushing syndrome" OR "Cushings Disease" OR "Cushings Syndrome" OR "Cushing's Disease" OR "Cushing's Syndrome" OR "Cushing Disease" OR "Cushing Syndrome" OR Cushing* OR "Hypercortisolism" OR Hypercortisol* OR "Pituitary ACTH Hypersecretion" OR "Inappropriate ACTH Secretion") AND ("transsphenoidal surgery" OR transsphenoid* OR trans-sphenoid* OR "Endoscopy"** OR **"Neuroendoscopy" OR Endoscop*** OR **Neuroendoscop* OR "Microsurgery" OR Microsurg* OR micro-surg* OR "microscopic surgery" OR microscopic surg*) AND ("Mortality" OR mortalit* OR "cause of death" OR "survival rate" OR "Peroperative Complication" OR "Surgical Injury" OR "Surgical Injuries" OR "Surgical Blood Loss" OR "Intraoperative Awareness" OR "Malignant Hyperthermia" OR "Postoperative Complication" OR "complication" OR "complications" OR "short term morbidity" OR "treatment failure" OR "hydrocortisone dependency" OR CSF leak* OR "meningitis" OR "bleeding" OR "diabetes insipidus" OR "SIADH" OR "anterior pituitary deficiency" OR "long term morbidity" OR "recurrent disease" OR "cardiovascular morbidity" OR "hypertension" OR "insulin resistance" OR "neuropsychiatric morbidity" OR "Morbidity" OR "morbidiy" OR "Treatment Failure" OR "liquorrhea" OR "Cerebrospinal Fluid Leak" OR "Cerebrospinal Fluid Leakage" OR "Meningitis" OR "Bleeding" OR hemorrhag* OR haemorrhag* OR "Diabetes Insipidus" OR "inappropriate vasopressin secretion" OR "Inappropriate ADH Syndrome" OR "anterior pituitary hormone deficiency" OR "hormone deficiency" OR "Recurrent Disease" OR "Hypertension" OR hypertens* OR "Insulin Resistance" OR "metabolic syndrome" OR neuropsychiat*)):ti,ab,kw**

**CENTRAL** (<http://archie.cochrane.org/?conferenceabstractredirectTo=http://crso.cochrane.org/login.php&key=4c7b65632dcd2>)
**(("Cushing disease" OR "Cushing syndrome" OR "Cushings Disease" OR "Cushings Syndrome" OR "Cushing's Disease" OR "Cushing's Syndrome" OR "Cushing Disease" OR "Cushing Syndrome" OR Cushing* OR "Hypercortisolism" OR Hypercortisol* OR "Pituitary ACTH Hypersecretion" OR "Inappropriate ACTH Secretion") AND ("transsphenoidal surgery" OR transsphenoid* OR trans-sphenoid* OR "Endoscopy"** OR **"Neuroendoscopy" OR Endoscop*** OR **Neuroendoscop* OR "Microsurgery" OR Microsurg* OR micro-surg* OR "microscopic surgery" OR microscopic surg*) AND ("Mortality" OR mortalit* OR "cause of death" OR "survival rate" OR "Peroperative Complication" OR "Surgical Injury" OR "Surgical Injuries" OR "Surgical Blood Loss" OR "Intraoperative Awareness" OR "Malignant Hyperthermia" OR "Postoperative Complication" OR "complication" OR "complications" OR "short term morbidity" OR "treatment failure" OR "hydrocortisone dependency" OR CSF leak* OR "meningitis" OR "bleeding" OR "diabetes insipidus" OR "SIADH" OR "anterior pituitary deficiency" OR "long term morbidity" OR "recurrent disease" OR "cardiovascular morbidity" OR "hypertension" OR "insulin resistance" OR "neuropsychiatric morbidity" OR "Morbidity" OR "morbidiy" OR "Treatment Failure" OR "liquorrhea" OR "Cerebrospinal Fluid Leak" OR "Cerebrospinal Fluid Leakage" OR "Meningitis" OR "Bleeding" OR hemorrhag* OR haemorrhag* OR "Diabetes Insipidus" OR "inappropriate vasopressin secretion" OR "Inappropriate ADH Syndrome" OR "anterior pituitary hormone deficiency" OR "hormone deficiency" OR "Recurrent Disease" OR "Hypertension" OR hypertens* OR "Insulin Resistance" OR "metabolic syndrome" OR neuropsychiat*))**

**Emcare** (<http://ovidsp.ovid.com/ovidweb.cgi?T=JS&NEWS=n&CSC=Y&PAGE=main&D=emcr>)
**((*"Cushing disease"/ OR *"Cushing syndrome"/ OR "Cushings Disease".ti,ab OR "Cushings Syndrome".ti,ab OR "Cushing's Disease".ti,ab OR "Cushing's Syndrome".ti,ab OR "Cushing Disease".ti,ab OR "Cushing Syndrome".ti,ab OR Cushing*.ti,ab OR "Hypercortisolism".ti,ab OR Hypercortisol*.ti,ab OR "Pituitary ACTH Hypersecretion".ti,ab OR "Inappropriate ACTH Secretion".ti,ab) AND ("transsphenoidal surgery"/ OR transsphenoid*.ti,ab OR trans-sphenoid*.ti,ab OR *"Endoscopy"/** OR ***"Neuroendoscopy"/ OR Endoscop*.ti,ab** OR **Neuroendoscop*.ti,ab OR *"Microsurgery"/ OR Microsurg*.ti,ab OR micro-surg*.ti,ab OR "microscopic surgery".ti,ab OR microscopic surg*.ti,ab) AND (exp "Mortality"/ OR mortalit*.ti,ab OR "cause of death".ti,ab OR "survival rate".ti,ab OR "Peroperative Complication"/ OR "Surgical Injury".ti,ab OR "Surgical Injuries".ti,ab OR "Surgical Blood Loss".ti,ab OR "Intraoperative Awareness".ti,ab OR "Malignant Hyperthermia".ti,ab OR exp "Postoperative Complication"/ OR "complication".ti,ab OR "complications".ti,ab OR "short term morbidity".ti,ab OR "treatment failure".ti,ab OR "hydrocortisone dependency".ti,ab OR CSF leak*.ti,ab OR "meningitis".ti,ab OR "bleeding".ti,ab OR "diabetes insipidus".ti,ab OR "SIADH".ti,ab OR "anterior pituitary deficiency".ti,ab OR "long term morbidity".ti,ab OR "recurrent disease".ti,ab OR "cardiovascular morbidity".ti,ab OR "hypertension".ti,ab OR "insulin resistance".ti,ab OR "neuropsychiatric morbidity".ti,ab OR "Morbidity"/ OR "morbidiy".ti,ab OR "Treatment Failure"/ OR "liquorrhea"/ OR "Cerebrospinal Fluid Leak".ti,ab OR "Cerebrospinal Fluid Leakage".ti,ab OR exp "Meningitis"/ OR exp "Bleeding" OR hemorrhag*.ti,ab OR haemorrhag*.ti,ab OR exp "Diabetes Insipidus"/ OR "inappropriate vasopressin secretion"/ OR "Inappropriate ADH Syndrome".ti,ab OR "anterior pituitary hormone deficiency".ti,ab OR exp "hormone deficiency"/ OR "Recurrent Disease"/ OR exp "Cardiovascular Disease"/ep OR exp "Hypertension"/ OR hypertens*.ti,ab OR exp "Insulin Resistance"/ OR "metabolic syndrome".ti,ab OR neuropsychiat*.ti,ab)) AND exp "Humans"/ NOT ((exp "case report"/ OR "case report".ti) NOT (exp "Clinical Trial"/ OR "series".ti OR "case study"/ OR exp "review"/ OR "review".ti))**

**LWW fulltext** (<http://ovidsp.ovid.com/ovidweb.cgi?T=JS&PAGE=main&MODE=ovidclassic&D=ovft>) **(("Cushing disease" OR "Cushing syndrome" OR "Cushings Disease" OR "Cushings Syndrome" OR "Cushing's Disease" OR "Cushing's Syndrome" OR "Cushing Disease" OR "Cushing Syndrome" OR Cushing* OR "Hypercortisolism" OR Hypercortisol* OR "Pituitary ACTH Hypersecretion" OR "Inappropriate ACTH Secretion").ti AND ("transsphenoidal surgery" OR transsphenoid* OR trans-sphenoid* OR "Endoscopy"** OR **"Neuroendoscopy" OR Endoscop*** OR **Neuroendoscop* OR "Microsurgery" OR Microsurg* OR micro-surg* OR "microscopic surgery" OR microscopic surg*).ti AND ("Mortality" OR mortalit* OR "cause of death" OR "survival rate" OR "Peroperative Complication" OR "Surgical Injury" OR "Surgical Injuries" OR "Surgical Blood Loss" OR "Intraoperative Awareness" OR "Malignant Hyperthermia" OR "Postoperative Complication" OR "complication" OR "complications" OR "short term morbidity" OR "treatment failure" OR "hydrocortisone dependency" OR CSF leak* OR "meningitis" OR "bleeding" OR "diabetes insipidus" OR "SIADH" OR "anterior pituitary deficiency" OR "long term morbidity" OR "recurrent disease" OR "cardiovascular morbidity" OR "hypertension" OR "insulin resistance" OR "neuropsychiatric morbidity" OR "Morbidity" OR "morbidiy" OR "Treatment Failure" OR "liquorrhea" OR "Cerebrospinal Fluid Leak" OR "Cerebrospinal Fluid Leakage" OR "Meningitis" OR "Bleeding" OR hemorrhag* OR haemorrhag* OR "Diabetes Insipidus" OR "inappropriate vasopressin secretion" OR "Inappropriate ADH Syndrome" OR "anterior pituitary hormone deficiency" OR "hormone deficiency" OR "Recurrent Disease" OR "Hypertension" OR hypertens* OR "Insulin Resistance" OR "metabolic syndrome" OR neuropsychiat*).af NOT ("case report" NOT ("Trial" OR "series" OR "review")).ti)**

**ScienceDirect fulltext** (<http://www.sciencedirect.com/science?_ob=MiamiSearchURL&_method=requestForm&_temp=all_boolSearch.tmpl&_acct=C000026638&_version=1&_urlVersion=1&_userid=530453&md5=d44bd9fa9076bb9b258a588b309be1e3>) **TITLE("Cushing disease" OR "Cushing syndrome" OR "Cushings Disease" OR "Cushings Syndrome" OR "Cushing's Disease" OR "Cushing's Syndrome" OR "Cushing Disease" OR "Cushing Syndrome" OR Cushing* OR "Hypercortisolism" OR Hypercortisol* OR "Pituitary ACTH Hypersecretion" OR "Inappropriate ACTH Secretion") AND TITLE("transsphenoidal surgery" OR transsphenoid* OR trans-sphenoid* OR "Endoscopy"** OR **"Neuroendoscopy" OR Endoscop*** OR **Neuroendoscop* OR "Microsurgery" OR Microsurg* OR micro-surg* OR "microscopic surgery" OR microscopic surg*) AND ("Mortality" OR mortalit* OR "cause of death" OR "survival rate" OR "Peroperative Complication" OR "Surgical Injury" OR "Surgical Injuries" OR "Surgical Blood Loss" OR "Intraoperative Awareness" OR "Malignant Hyperthermia" OR "Postoperative Complication" OR "complication" OR "complications" OR "short term morbidity" OR "treatment failure" OR "hydrocortisone dependency" OR CSF leak* OR "meningitis" OR "bleeding" OR "diabetes insipidus" OR "SIADH" OR "anterior pituitary deficiency" OR "long term morbidity" OR "recurrent disease" OR "cardiovascular morbidity" OR "hypertension" OR "insulin resistance" OR "neuropsychiatric morbidity" OR "Morbidity" OR "morbidiy" OR "Treatment Failure" OR "liquorrhea" OR "Cerebrospinal Fluid Leak" OR "Cerebrospinal Fluid Leakage" OR "Meningitis" OR "Bleeding" OR hemorrhag* OR haemorrhag* OR "Diabetes Insipidus" OR "inappropriate vasopressin secretion" OR "Inappropriate ADH Syndrome" OR "anterior pituitary hormone deficiency" OR "hormone deficiency" OR "Recurrent Disease" OR "Hypertension" OR hypertens* OR "Insulin Resistance" OR "metabolic syndrome" OR neuropsychiat*) NOT TITLE("case report")**

**Wiley fulltext** (<http://onlinelibrary.wiley.com/advanced/search>)
**TITLE**

**("Cushing disease" OR "Cushing syndrome" OR "Cushings Disease" OR "Cushings Syndrome" OR "Cushing's Disease" OR "Cushing's Syndrome" OR "Cushing Disease" OR "Cushing Syndrome" OR Cushing* OR "Hypercortisolism" OR Hypercortisol* OR "Pituitary ACTH Hypersecretion" OR "Inappropriate ACTH Secretion")**

**AND TITLE**

**("transsphenoidal surgery" OR transsphenoid* OR trans-sphenoid* OR "Endoscopy"** OR **"Neuroendoscopy" OR Endoscop*** OR **Neuroendoscop* OR "Microsurgery" OR Microsurg* OR micro-surg* OR "microscopic surgery" OR microscopic surg*)**

**AND**

**("Mortality" OR mortalit* OR "cause of death" OR "survival rate" OR "Peroperative Complication" OR "Surgical Injury" OR "Surgical Injuries" OR "Surgical Blood Loss" OR "Intraoperative Awareness" OR "Malignant Hyperthermia" OR "Postoperative Complication" OR "complication" OR "complications" OR "short term morbidity" OR "treatment failure" OR "hydrocortisone dependency" OR CSF leak* OR "meningitis" OR "bleeding" OR "diabetes insipidus" OR "SIADH" OR "anterior pituitary deficiency" OR "long term morbidity" OR "recurrent disease" OR "cardiovascular morbidity" OR "hypertension" OR "insulin resistance" OR "neuropsychiatric morbidity" OR "Morbidity" OR "morbidiy" OR "Treatment Failure" OR "liquorrhea" OR "Cerebrospinal Fluid Leak" OR "Cerebrospinal Fluid Leakage" OR "Meningitis" OR "Bleeding" OR hemorrhag* OR haemorrhag* OR "Diabetes Insipidus" OR "inappropriate vasopressin secretion" OR "Inappropriate ADH Syndrome" OR "anterior pituitary hormone deficiency" OR "hormone deficiency" OR "Recurrent Disease" OR "Hypertension" OR hypertens* OR "Insulin Resistance" OR "metabolic syndrome" OR neuropsychiat*) NOT TITLE("case report")**
