# Supplementary material for: Endoscopic vs. microscopic transsphenoidal surgery for Cushing’s disease: a systematic review and meta-analysis
Source: Pituitary. 2018 May 16;21(5):524–34. doi: 10.1007/s11102-018-0893-3 (PMC6132967; doi:10.1007/s11102-018-0893-3)
Supplement: Supplementary file 2 — Supplementary material 2 (DOCX 33 KB) [file 11102_2018_893_MOESM2_ESM.docx]

**Pituitary
Endoscopic vs. microscopic transsphenoidal surgery for Cushing’s disease: a systematic review and meta-analysis.**

Leonie H.A. Broersen^1,2^, Nienke R. Biermasz^1,2^, Wouter R. van Furth^2,3^, Friso de Vries^1,2^, Marco J.T. Verstegen^2,3^, Olaf M. Dekkers^1,4^, Alberto M. Pereira^1,2^

^1^Department of Medicine, division of Endocrinology, Leiden University Medical Centre, Albinusdreef 2, 2333 ZA, Leiden, The Netherlands
^2^Center for Endocrine Tumors Leiden (CETL), Leiden University Medical Center, Albinusdreef 2, 2333 ZA, Leiden, The Netherlands
^3^Department of Neurosurgery, Leiden University Medical Centre, Leiden, Albinusdreef 2, 2333 ZA, The Netherlands
^4^Department of Clinical Epidemiology, Leiden University Medical Center, Leiden, Albinusdreef 2, 2333 ZA, The Netherlands

Corresponding author: L.H.A. Broersen, [L.H.A.Broersen@lumc.nl](mailto:L.H.A.Broersen@lumc.nl), +31 (0)71-5263082

Online Resource 2: study characteristics.

|  | **Calendar year of surgery** | **Center (city, country)** | **Age, mean (range) in years** | **Gender, female/male** | **Duration of follow-up, mean (range) in years** |
| --- | --- | --- | --- | --- | --- |
| ***Microscopic surgery*** |  |  |  |  |  |
| Acebes 2007 | 1997-2005 | Barcelona, Spain | 41.5 (9-86)^a^ | 39/5 | 4.1 (1.6-8.5) |
| Alahmadi 2013 | 2000-2010 | Toronto, Canada | 50 (20-69)^b^ | 31/11^b^ | 2.8 (0.3-8.5)^b^ |
| Alexandraki 2013 | 1969-2001 | London, UK | 39.2 (7-95)^b^ | 102/29^b^ | 15.4 (6-36)^b^ |
| Alwani 2010 | 1991-2006 | Rotterdam, the Netherlands | 40.8 (SE 1.46) | 63/16 | 7 (0.6-10.1)^a^ |
| Ammini 2011 | 2000-2009 | New Delhi, India | 25 (8-63)^a,c^ | 82/39^c^ | (1.5-11)^c^ |
| Arnott 1990 | 1983-1987 | Fitzroy, Australia | (11-65) | 19/9 | 1.9 (0.3-4.7) |
| Asuzu 2017 | 2003-2016 | Bethesda, MD, USA | Not reported | Not reported | Not reported |
| Atkinson 2008 | 1995-2003 | Rochester, NY, USA | 45.0 (27-69) | 17/4 | 1.0 (0.3-11.0)^a^ |
| Bakiri 1996 | Not reported | Suresnes, France | 29.64 (10-55) | 34/16 | 6.0 (2.1-18.3)^a^ |
| Barbetta 2001 | Not reported | Milan, Italy | (13-70) | 56/12 | 4.8 (1.0-21.0)^a^ |
| Barbot 2013 | 2001-2009 | Padua, Italy | 38 (13-67)^a^ | 48/9 | 6.4 (2.0-10.3)^a^ |
| Barzaghi 2007 | 1990-2004 | Milan, Italy | 43.7 (SE 0.4)^c^ | 746/494^c^ | Not reported |
| Bay 1988 | 1976-1986 | Cleveland, OH, USA | 41 (17-65) | 31/5 | 3.8 (0.3-10.0) |
| Bigos 1980 | 1963-1977 | Montreal, Canada | (13-58) | 19/5 | 1.0 (0.1-15.0)^a^ |
| Blevins 1998 | 1972-1995 | Atlanta, GA, USA | 38.6 | Ratio microadenoma 4.5:1, ratio macroadenoma 10:1 | (0.7-18.2) |
| Burkhardt 2013 | 2009-2010 | Hamburg, Germany | 42 (11-73) | 32/10 | Not reported |
| Chandler 2016 | 1980-2012 | Ann Arbor, MI, USA | 39.9 (8-76) | 215/60 | 6.7 (1-29) |
| Chee 2001 | 1980-1997 | Newcastle upon Tyne, UK | 37.3 (12-69) | 45/16 | 7.3 (0.6-17.6)^a^ |
| Chen 2003 | 1973-1993 | Los Angeles, CA and Salt Lake City, UT, USA | (8-57)^e^ | 104/48^e^ | Not reported |
| Cheng 2011 | 2003-2009 | Shanghai, China | 33.8 (11-71)^c^ | 39/20^c^ | 2.3 (0.4-5.3)^c^ |
| Ciric 2012 | 1970-2010 | Evanston, IL, USA | (8-80)^e^ | 109/27^e^ | (0-33) |
| D’Haens 2009 | 1995-2001 | Brussels, Belgium | 35 (10-68)^c^ | 44/16^c^ | 5.1 (0.1-12.0) |
| Donofrio 2016 | 2007-2014 | Milan, Italy | 43.6 (9.0-73.2) | 105/37 | Not reported |
| Erem 2003 | 1983-2000 | Istanbul, Turkey | 35.4 (16-63)^e^ | 25/3^e^ | 2.1 (±1.5) |
| Esposito 2006 | 1998-2004 | Los Angeles, CA, USA | 39 (21-70) | 37/3 | 2.8 (1.2-5.4) |
| Flitsch 2003 | 1990-1996 | Hamburg, Germany | 35 (±16.2) | 111/36 | 5.1 (1.2-10.3) |
| Gazioglu 2008 | 2000-2006 | Istanbul, Turkey | 39.6 (17-62) | 23/3 | Not reported |
| Gsponer 1999 | 1984-1997 | Lausanne, Switzerland | 44.6 (18-68) | 14/1 | Not reported |
| Guilhaume 1988 | 1978-1985 | Paris, France | 38.3 (19-69) | 46/18 | 2 (0.3-7)^a^ |
| Hammer 2004 | 1975-1998 | San Francisco, CA, USA | 37 (18-72)^a,b^ | 239/50^b^ | 11.1 (0.6-24.1)^a,b,e^ |
| Hardy 1979 | Not reported | Montreal, Canada | Not reported | Not reported | Not reported |
| Hofmann 2008 | 1971-2004 | Erlangen, Germany | 39.4 (5-77) | 325/101 | 6.0 (0.3-25.0) |
| Honegger 2012 | 1998-2011 | Freiburg and Tübingen, Germany | 46 (12-76) | 61/22 | 3.2 (0.3-13) |
| Hoybye 2004 | 1990-1999 | Stockholm, Sweden | 40 (13-74) | 26/8 | 6 (1-12) |
| Huan 2014 | 2002-2011 | Jinan, China | 33.6 (17-75) | 59/25 | 3.8 (1.1-10.1)^a^ |
| Imaki 2001 | 1977-1997 | Tokyo, Japan | 37.6 (SD 13) | 42/7 | 7.3 (2.0-21.1) |
| Inder 2003 | 1984-2000 | Christchurch, New Zealand | 38 (11-66)^b^ | 11/5^b^ | 3.4 (1-9)^b^ |
| Jagannathan 2009 | 1990-2007 | Charlottesville, VA, USA | 32.5 (4.7-77)^e^ | 199/62^e^ | 7.0 (1.0-17.9)^e^ |
| Jehle 2008 | 1987-2005 | New York, NY, USA | 40.9 (SD 13.7) | 159/34 | 5.2 |
| Knappe 2011 | Not reported | Minden and Wiesbaden, Germany | 42.8 (24-71)^c^ | 14/4^c^ | 1.1 (0.3-2.7)^c^ |
| Kristof 2002 | 1990-1999 | Bonn, Germany | 32^a^ | 20/7 | 3.7^c^ |
| Kurosaki 2000 | 1997-1999 | Hamburg, Germany | 37.2 (9-67) | 40/11 | (0.5-3) |
| Lampropoulos 2013 | 2004-2011 | Marousi, Greece | 46.6 (SD 11.9) | 21/2 | 3.6 (SD 2)^c^ |
| Lüdecke 1985 | 1976-1984 | Hamburg, Germany | Not reported | Not reported | Not reported |
| Lüdecke 1991 | 1987-1990 | Hamburg, Germany | Not reported | Not reported | Not reported |
| Mampalam 1988 | 1974-1986 | San Francisco, CA, USA | 35 (6-71) | 179/42 | 3.8 (1-13) |
| Mehrazin 2004 | 1992-2000 | Tehran, Iran | 21.5 (9-34) | 8/3 | 5 (0.5-8) |
| Mortini 2005 | 1990-2002 | Milan, Italy | 38.4 (SE 0.8) | 214/48 | Not reported |
| Nakane 1987 | 1977-1984 | Nagoya, Japan | 34 (9-58) | 70/30 | 3.2 (SD 2.0) |
| Nemergut 2005 | 1995-2001 | Charlottesville, VA, USA | Not reported | Not reported | Not reported |
| Norris 1997 | 1984-1995 | London, UK | 36.0 (7.6-67) | 71/29 | 5.7 (0.3-10.9) |
| Patil 2008 | 1992-2006 | Charlottesville, VA, USA | 39.6 (±15) | 167/48 | 3.8 (0.5-13.8) |
| Patil 2008 (repeat surgery only) | 1992-2006 | Charlottesville, VA, USA | 40.3 (17.1-63.0) | 26/10 | 3.0 (0.2-10.5) |
| Petruson 1997 | 1984-1993 | Gothenburg, Sweden | 44 (13-75) | 26/5 | 4.5 (1-10)^a^ |
| Pieters 1989 | 1981-1988 | Nijmegen, the Netherlands | 33.6 (SD 6) | 22/5 | 4.5 (1.5-7.5)^a^ |
| Pikkarainen 1999 | 1981-1994 | Helsinki, Finland | 44.6 (23-79)^c^ | 38/5 | 7.4 (0-15)^c^ |
| Pimentel-Filho 2005 | Not reported | São Paulo, Brazil | 31.1 | 13/4 | Not reported |
| Post 1995 | 1975-1992 | Cape Town, South Africa | 33.6 (13-64) | 26/8 | 5.8 (1.2-15) |
| Potts 2014 | 2000-2008 | San Francisco, CA, USA | 40.4 (SD 14.8) | 80/11 | 1 (0.02-9.4)^a,e^ |
| Powell 2017 | 2000-2013 | Tashkent, Uzbekistan | (3-59)^c^ | Ratio 2:1^c^ | Not reported |
| Rollin 2007 | 1989-unknown | Porto Alegre, Brazil | 37 (12-68) | 83/25 | 6 (0.2-18.3) |
| Salassa 1978 (abstract only) | Not reported | Rochester, MN, USA | Not reported | Not reported | Not reported |
| Semple, C.G. 1984 | 1976-1982 | Glasgow, UK | 29.8 (15-49) | 8/2 | 3.3 (1.3-6.3)^b^ |
| Semple, P.L. 1999 | 1992-1997 | Charlottesville, VA, USA | 38.5 (6-78) | 83/22 | Not reported |
| Shah 2006 | Not reported | Mumbai, India | Not reported | Not reported | Not reported |
| Shimon 2002 | 1990-2000 | Tel Hashomer, Israel | 39.0 (8-72) | 71/11 | 4.2 (SD 2.9) |
| Shirvani 2016 | 1997-2012 | Tehran, Iran | 31.4 (7-65) | 73/23 | 3.7 (3-13) |
| Sonino 1996 | 1975-1995 | Padua, Italy | 37.5 (11-71)^c^ | 124/38^c^ | 7.3 (2-18)^c^ |
| Sudhakar 2004 | 1996-2001 | Cardiff, UK | Not reported | Not reported | Not reported |
| Swearingen 1999 | 1978-1996 | Boston, MA, USA | 38 (8-76) | 129/32 | 8.7 (1-20) |
| Valderrabano 2014 (repeat surgery only) | 1982-2009 | Madrid, Spain | 37.1 (14-64) | 21/5 | 11.6 (5.2-15.3)^e^ |
| Vallette-Kasic 2000 | 1989-1998 | Marseille, France | 39 (13-62) | 49/4 | 3.2 (2.0-10.0) |
| Witek 2012 | 2005-2009 | Warsaw, Poland | 36.3 (17-57) | 30/6 | 2.4 (1.5-3.0) |
| Witek 2016 | 2013-2015 | Warsaw, Poland | 42.4 (17-79) | 32/8 | 0.5 |
| Yap 2002 | 1969-1998 | Oxford, UK | 39.1 (14-82) | 78/19 | 7.7 (0.5-29) |
| ***Endoscopic surgery*** |  |  |  |  |  |
| Alahmadi 2013 | 2000-2010 | Toronto, Canada | 50 (20-69)^b^ | 31/11^b^ | 2.8 (0.3-8.5)^b^ |
| Atkinson 2008 | 1995-2003 | Rochester, MN, USA | 43.8 (19.0-70.0) | 16/5 | 2.5 (0.3-8.7)^a^ |
| Berker 2013 | 2006-2012 | Ankara, Turkey | 38.74 (±13.01)^e^ | 79/11^e^ | 2.7 (0.4-6.3)^a,e^ |
| Cebula 2017 | 2008-2013 | Suresnes, France | 42 (18-83) | 188/42 | 1.8 (0.1-5.8)^a^ |
| Cheng 2011 | 2003-2009 | Shanghai, China | 37.2 (13-69)^c^ | 37/31^c^ | 1.9 (0.3-4.7)^c^ |
| Dehdashti 2007 | 2004-2007 | Toronto, Canada | 42 (±12.5) | 19/6 | 1.4 (0.2-2.8)^a^ |
| Dehdashti 2008 | 2004-2007 | Toronto, Canada | 49.9 (20-78)^c^ | 109/91^c^ | 1.6 (0.4-2.7)^a^ |
| D’Haens 2009 | 2001-2007 | Brussels, Belgium | 37 (10-70)^c^ | 41/19^c^ | 1.5 (0.1-6.3) |
| Frank 2006 | 1998-2004 | Bologna, Italy | 41 (12-80) | 32/24 | 4.5 (1.3-7.8)^a,c^ |
| Gondim 2010 | 1998-2007 | Fortaleza, Brazil | 42.51 (13-79)^c^ | 126/102^c^ | 5.1 (0.7-11)^c^ |
| Hofstetter 2011 | 2004-2010 | New York, NY, USA | 53.8 (SE 3.8) | 13/5 | 2.1 (SE 0.4) |
| Hwang 2009 | 1996-2006 | Seoul, South Korea | 38.7 (13-73)^b^ | 25/5^e^ | 3.4 (0.8-8.4) |
| Jho 2001 | 1993-1999 | Pittsburgh, PA, USA | 43 (14-88)^a,c^ | 90/70^c^ | Not reported |
| Kabil 2005 | 1998-2004 | Los Angeles, CA, USA | 46 (16-75)^c^ | 189/111^c^ | 3.2 (0.2-5.8)^c^ |
| Kuo 2015 | 2000-2014 | Taipei, Taiwan | 41.0 (SD 13.0) | 38/2 | 3.4 (SD 2.5) |
| Leach 2010 | 2005-2007 | Salford, UK | 51 (18-85)^c^ | 55/70^c^ | 1.5 (0.8-3.3)^c^ |
| Mamelak 2012 | 2006-2011 | Los Angeles, CA, USA | 51.6 (±12)^c^ | 146/130^c^ | 3.3 (0.5-5.5) |
| Masopust 2017 | 2008-unknown | Prague, Czech Republic | 40 (8-78) | 30/11 | (0.5-2.8) |
| Paluzzi 2014 | 2002-2011 | Pittsburgh, PA, USA | Not reported | Not reported | 3.1 (0.3-9.5)^c^ |
| Rudnik 2007 (abstract only) | Not reported | Katowice, Poland | 42.6 (11-77)^c^ | 51/19^c^ | 2.8 (0.6-4.7)^c^ |
| Sarkar 2016 | 2009-2014 | Vellore, India | 31.9 (SD 9.6) | 51/13 | 1.7 (0.3-5.3) |
| Senior 2008 | 2000-2007 | Chapel Hill, NC, USA | 46 (12-88)^c^ | 96/80^c^ | (0.3-7.3)^c^ |
| Shin 2015 | 2002-2013 | Pittsburgh, PA, USA | 44 (19.0-66.7)^a^ | 39/11 | 3.1 (0.2-12.9) |
| Starke 2013 | 2004-2011 | Charlottesville, VA and Boston, MA, USA | 49 (14-63) | 52/9 | 2.3 (1.0-6.0)^e^ |
| Torales 2014 | 2005-2012 | Barcelona, Spain | 55.7 (18-82)^c^ | 70/51^c^ | 4.6 (1.1-8.6)^c^ |
| Wagenmakers 2013 | 1998-2011 | Nijmegen, the Netherlands | 42.3 (SD 14.9) | 62/24 | 5.9 (0.4-13.7) |

^a^Median (range).
^b^Not reported separately per type of surgery.
^c^Not specified for Cushing’s disease only.
^d^Median (interquartile range).
^e^Does not exactly cover included population.
