# Supplementary material for: Endoscopic vs. microscopic transsphenoidal surgery for Cushing’s disease: a systematic review and meta-analysis
Source: Pituitary. 2018 May 16;21(5):524–34. doi: 10.1007/s11102-018-0893-3 (PMC6132967; doi:10.1007/s11102-018-0893-3)
Supplement: Supplementary file 3 — Supplementary material 3 (DOCX 45 KB) [file 11102_2018_893_MOESM3_ESM.docx]

**Pituitary
Endoscopic vs. microscopic transsphenoidal surgery for Cushing’s disease: a systematic review and meta-analysis.**

Leonie H.A. Broersen^1,2^, Nienke R. Biermasz^1,2^, Wouter R. van Furth^2,3^, Friso de Vries^1,2^, Marco J.T. Verstegen^2,3^, Olaf M. Dekkers^1,4^, Alberto M. Pereira^1,2^

^1^Department of Medicine, division of Endocrinology, Leiden University Medical Centre, Albinusdreef 2, 2333 ZA, Leiden, The Netherlands
^2^Center for Endocrine Tumors Leiden (CETL), Leiden University Medical Center, Albinusdreef 2, 2333 ZA, Leiden, The Netherlands
^3^Department of Neurosurgery, Leiden University Medical Centre, Leiden, Albinusdreef 2, 2333 ZA, The Netherlands
^4^Department of Clinical Epidemiology, Leiden University Medical Center, Leiden, Albinusdreef 2, 2333 ZA, The Netherlands

Corresponding author: L.H.A. Broersen, [L.H.A.Broersen@lumc.nl](mailto:L.H.A.Broersen@lumc.nl), +31 (0)71-5263082

Online Resource 3: risk of bias assessment.

|  | **Loss to follow-up, N (%)** | **Inclusion of patients** | **Criteria for diagnosis of Cushing’s disease (used tests)** | **Criteria for main study outcomes (tests used for determination of remission; time of tests)** | **Co-interventions** |
| --- | --- | --- | --- | --- | --- |
| ***Microscopic surgery*** |  |  |  |  |  |
| Acebes 2007 | Not reported | Consecutive | Mentioned (serum cortisol, 24-h UFC, ACTH, MRI, IPSS) | Mentioned (basal and ACTH-stimulated plasma cortisol, 24-h UFC, remission of symptoms; within 6 months) | No co-interventions |
| Alahmadi 2013 | Not reported | Consecutive | Mentioned (clinical criteria, 1 mg and 8 mg dexamethasone test, 24-h UFC, midnight salivary cortisol, serum cortisol, ACTH, MRI, IPSS) | Mentioned (24-h UFC, 1 mg dexamethasone test, or dependent on cortisol replacement; 3 and 6 months postoperatively) | Not reported |
| Alexandraki 2013 | 26 patients had a follow-up <6 years (16.6%) | Minimum follow-up of six years | Mentioned (clinical criteria, circadian rhythm, low and high dose dexamethasone test, CRH test, IPSS) | Mentioned (postoperative cortisol, postoperative cortisol insufficiency with need for replacement, normalization of hypercortisolism, or low dose dexamethasone test; within 14 days, at least 12h after replacement dose) | No co-interventions |
| Alwani 2010 | 6 (7.5%) | Consecutive | Mentioned (clinical criteria, 24-h UFC, 1 mg dexamethasone test, circadian rhythm, ACTH, serum cortisol, MRI, IPSS) | Mentioned (steroid replacement >6 months, 1 mg dexamethasone test, UFC; 7-10d postoperatively, 72 h after replacement dose) | 60x presurgical treatment with medication |
| Ammini 2011 | Not reported | Consecutive | Mentioned (clinical criteria, 1 mg dexamethasone test, salivary cortisol, ACTH, high dose dexamethasone test, MRI, IPSS) | Mentioned (serum cortisol ; 1d or 1 week postoperatively, 24h after replacement dose) | Not reported |
| Arnott 1990 | Not reported | Consecutive | Mentioned (diurnal variation plasma cortisol, UFC, low dose dexamethasone test, high dose dexamethasone test, metyrapone test, CT, IPSS) | Mentioned (UFC, steroid replacement required, clinical symptoms; 1 week - 1 month postoperatively) | 1x radiotherapy |
| Asuzu 2017 | Not reported | Consecutive (for repeat surgery only results for early reoperation reported) | Mentioned (late night salivary cortisol, 24-h UFC, low dose dexamethasone test, midnight serum cortisol, ACTH, 24-h urine 17OHS, 8 mg dexamethasone test, CRH test, MRI/CT, IPSS) | Mentioned | Not reported |
| Atkinson 2008 | Not reported | Only micro-adenoma, follow-up >3 months | Not mentioned | Mentioned (postoperative hypocortisolism) | No co-interventions |
| Bakiri 1996 | 3 (6.0%) | Consecutive | Mentioned (clinical criteria, diurnal rhythm, UFC, low dose dexamethasone test, ACTH, high dose dexamethasone test, MRI) | Mentioned (clinical and biochemical signs, circadian rhythm, low dose dexamethasone suppression test) | 3x unilateral adrenalectomy, 1x radiotherapy |
| Barbetta 2001 | Not reported | Minimum follow-up of 12 months | Mentioned (clinical criteria, UFC, ACTH, serum cortisol, low dose dexamethasone test, high dose dexamethasone test, CRH test, desmopressin test, MRI/CT) | Not mentioned | Not reported |
| Barbot 2013 | Not reported | Minimum follow-up of 24 months | Mentioned (24-h UFC, late night serum or salivary cortisol, low dose dexamethasone test, ACTH, high dose dexamethasone test, CRH test, MRI, IPSS) | Mentioned (steroid replacement, serum cortisol, UFC, low dose dexamethasone test, clinical features; 6 months) | Not reported |
| Barzaghi 2007 | Not reported | Consecutive | Mentioned (MRI/CT, histology, IHC) | Mentioned | Not reported |
| Bay 1988 | Not reported | Consecutive | Mentioned (clinical criteria, plasma cortisol, diurnal rhythm, 24-h UFC, low dose dexamethasone test, high dose dexamethasone test, ACTH, CT) | Mentioned (clinical symptoms and signs, laboratory correction of hypercortisolism) | Not reported |
| Bigos 1980 | Not reported | Consecutive | Mentioned (clinical criteria, 24-h UFC or 17OHCS, diurnal rhythm, 8 mg dexamethasone suppression test, radiology sella) | Mentioned | 1x radiotherapy |
| Blevins 1998 | 20 patients had a follow-up <6 months (17.2%) | Minimum follow-up of 6 months | Mentioned (clinical criteria, UFC, ACTH, dynamic tests (dexamethasone, metyrapone, CRH)) | Mentioned (postoperatively: cortisol, 24-h UFC, requiring steroid replacement for any period; after 6 months: dexamethasone test, plasma cortisol, UFC) | Not reported |
| Burkhardt 2013 | Not reported | Consecutive | Not mentioned | Mentioned (morning cortisol; within 1 week postoperatively) | Not reported |
| Chandler 2016 | Not reported | Consecutive | Not mentioned | Mentioned (cortisol, UFC) | Not reported |
| Chee 2001 | 2 (3.3%) | Consecutive | Mentioned (clinical criteria, UFC, diurnal rhythm, low dose dexamethasone test, high dose dexamethasone test, ACTH, CRH, CT/MRI, IPSS) | Mentioned (biochemical reversal of hypercortisolism, diurnal circadian rhythm, clinical features, low dose dexamethasone test; 6-8 weeks postoperatively after discontinuation of replacement cortisol) | Not reported |
| Chen 2003 | 10 (6.2%) | Only patients with adenoma found during exploration | Mentioned (diurnal rhythm, 24-h UFC, ACTH, low dose dexamethasone test, high dose dexamethasone test, CT/MRI, IPSS) | Mentioned (low dose dexamethasone test; day 3 postoperatively) | Not reported |
| Cheng 2011 | Not reported | Consecutive | Mentioned (serum cortisol, 24-h UFC, ACTH, MRI) | Mentioned (24-h UFC, morning serum cortisol, ACTH) | Not reported |
| Ciric 2012 | 6 (4.4%) | Consecutive | Mentioned (clinical criteria, 24-h UFC, late night salivary cortisol, plasma cortisol, low dose dexamethasone test, circadian rhythm, ACTH, MRI, IPSS) | Mentioned (plasma cortisol, symptomatic adrenal insufficiency requiring steroid replacement; 1d postoperatively) | 1x presurgical treatment with medication |
| D’Haens 2009 | Not reported | Consecutive | Mentioned (ACTH, 24-h UFC, circadian rhythm plasma cortisol, 1 mg dexamethasone test, MRI, IPSS) | Mentioned (serum ACTH and cortisol, 24-h UFC; at least 6 weeks postoperatively) | Not reported |
| Donofrio 2016 | Not reported | Consecutive | Mentioned (clinical criteria, 24-h UFC, circadian rhythm, 1 mg dexamethasone test, ACTH, MRI, IPSS) | Mentioned | No presurgical treatment with medication |
| Erem 2003 | 3 (10.7%) | Consecutive | Mentioned (clinical criteria, serum cortisol, 24-h UFC, low dose dexamethasone test, high dose dexamethasone test, CT/MRI, ACTH) | Mentioned (cortisol; 1-2d postoperatively) | Not reported |
| Esposito 2006 | 0 (0%) | Consecutive | Mentioned (clinical criteria, diurnal rhythm, ACTH, low dose dexamethasone test, high dose dexamethasone test, 24-h UFC, IPSS, MRI) | Mentioned (morning cortisol, necessitating steroid replacement; 1-2d postoperatively) | 1x radiotherapy |
| Flitsch 2003 | Not reported | Primary surgery, follow-up >12 months | Mentioned (hypercortisolism, ACTH, high dose dexamethasone test, CRH test, MRI) | Mentioned (perioperative decline of cortisol to (sub)normal levels; within 1 week postoperatively) | 19x presurgical treatment with medication |
| Gazioglu 2008 | 1 (3.8%) | Only patients with negative MRI or adenoma <6 mm | Mentioned (basal cortisol, ACTH, 24-h UFC, diurnal rhythm, low and high dose dexamethasone test, MRI, IPSS) | Mentioned (basal cortisol, 1 mg dexamethasone test, ACTH; within 1 week postoperatively) | Not reported |
| Gsponer 1999 | Not reported | Consecutive | Mentioned (ACTH, circadian rhythm, 1 mg dexamethasone test, long dexamethasone test, UFC, CRH test, IPSS, CT/MRI) | Not mentioned | Not reported |
| Guilhaume 1988 | 3 (4.7%) | Consecutive | Mentioned (clinical criteria, urinary 17OHS, cortisol, diurnal rhythm, low dose dexamethasone test, high dose dexamethasone test, metyrapone test, ACTH, lipotropin, X-sella/CT) | Mentioned (basal urinary cortisol, diurnal plasma cortisol, lysine-8 vasopressine test, ACTH, lipotropin; 3-6 months postoperatively) | 1x partial adrenalectomy, 24x presurgical treatment with medication, 3x radiotherapy |
| Hammer 2004 | Not reported | Consecutive | Mentioned (clinical criteria, laboratory, MRI, IPSS) | Mentioned (basal or dexamethasone-suppressed plasma cortisol, UFC, clinical features, no additional therapy; within 1 week postoperatively) | Not reported |
| Hardy 1979 | Not reported | Consecutive | Not mentioned | Mentioned (24-h UFC, 2 mg/d dexamethasone test) | Not reported |
| Hofmann 2008 | 64 in total (23 after first follow-up) (15.0%) | Primary surgery, at least follow-up at 3 months | Mentioned (clinical criteria, ACTH, cortisol, 2 mg dexamethasone test, high dose dexamethasone test, CRH test, IPSS, UFC, diurnal rhythm, X-sella/CT) | Mentioned (2 mg dexamethasone test; 3 months postoperatively) | Not reported |
| Honegger 2012 | Not reported | Consecutive | Mentioned (ACTH, CRH test, high dose dexamethasone test, IPSS, MRI) | Mentioned (24-h UFC, low dose dexamethasone test, clinical signs; 4 weeks – 3 months postoperatively) | Not reported |
| Hoybye 2004 | Not reported | Consecutive | Mentioned (clinical criteria, MRI, IPSS, circadian rhythm, ACTH, 24-h UFC, 1 mg dexamethasone test, low and high dose dexamethasone test) | Mentioned (clinical remission, serum cortisol, ACTH, 24-h UFC, 1 mg dexamethasone test; 8-12 weeks postoperatively) | No presurgical treatment with medication |
| Huan 2014 | Not reported | Primary surgery, no co-interventions | Mentioned (clinical criteria, CT/MRI, cortisol, diurnal rhythm, 24-h UFC, low and high dose dexamethasone test) | Mentioned (return of hormone levels to normal range without further treatment; within 3 months postoperatively) | No co-interventions |
| Imaki 2001 | 0 (0%) | Unclear | Mentioned (clinical criteria, UFC, circadian rhythm, low dose dexamethasone test, high dose dexamethasone test, CRH test, ACTH, IPSS, MRI) | Mentioned (regression of symptoms, ACTH, serum cortisol, UFC, 1 mg dexamethasone test; 3-8 weeks postoperatively) | Pre-surgical: 3x subtotal adrenalectomy, 3x radiotherapy, 1x medication; post-surgical: 19x radiotherapy |
| Inder 2003 | 1 (6.3%) | Consecutive | Mentioned (clinical criteria, 24-h UFC, diurnal rhythm, 1 mg dexamethasone test, ACTH, CT/MRI, CRH test, high dose dexamethasone test, IPSS) | Mentioned (morning plasma cortisol; within 14 days postoperatively) | 1x radiotherapy, 1x presurgical treatment with medication, 1x unilateral adrenalectomy |
| Jagannathan 2009 | 0 (0%) | Consecutive | Mentioned (ACTH, diurnal rhythm, 24-h UFC, dexamethasone test, CRH test, MRI, IPSS) | Mentioned (UFC, morning serum cortisol; within 1.5 week postoperatively, after stopping replacement therapy for at least 3 days) | Not reported |
| Jehle 2008 | Not reported | Consecutive | Mentioned (UFC, ACTH, serum cortisol, low and high dose dexamethasone test, salivary cortisol, MRI/CT, IPSS) | Mentioned (UFC, serum cortisol, need for cortisol substitution, 1 mg dexamethasone test) | Not reported |
| Knappe 2011 | Not reported | Consecutive | Mentioned (diurnal rhythm, 24-h UFC, ACTH, low dose dexamethasone test, high dose dexamethasone test, CRH test, MRI, IPSS) | Mentioned (serum cortisol; 1-2 days postoperatively) | Not reported |
| Kristof 2002 | 0 (0.0%) | Consecutive | Mentioned (ACTH, cortisol, 24-h UFC, 2 mg dexamethasone test, CRH test, MRI) | Mentioned (basal cortisol, 24-h UFC, 2 mg dexamethasone test; early and 3 months postoperatively) | Not reported |
| Kurosaki 2000 | Not reported | Microadenomas only | Mentioned (ACTH, high dose dexamethasone test, CRH test, IPSS, MRI) | Mentioned (cortisol, ACTH, need for replacement of cortisol; 6 months – 3y postoperatively) | Not reported |
| Lampropoulos 2013 | Not reported | Consecutive | Mentioned (ACTH, cortisol, 24-h UFC, MRI/CT) | Mentioned (morning cortisol, requiring substitution within 48-h, low dose dexamethasone test, UFC; 6 weeks postoperatively) | Not reported |
| Lüdecke 1985 | 22 (25.0%) | Consecutive | Not mentioned | Mentioned (morning cortisol diurnal pattern, low dose dexamethasone test; 6-8d postoperatively) | Not reported |
| Lüdecke 1991 | Not reported | Consecutive | Mentioned (CRH test, high dose dexamethasone test, ACTH, IPSS, CT/MRI) | Mentioned | Not reported |
| Mampalam 1988 | 2 (0.9%) | Consecutive | Mentioned (morning and evening plasma cortisol, 24-h UFC, ACTH, low dose dexamethasone test, high dose dexamethasone test, CT/MRI, IPSS) | Mentioned (resolution of clinical signs, plasma cortisol, ACTH, low dose dexamethasone test) | 12x radiotherapy, 5x bilateral adrenalectomy, 1x craniotomy |
| Mehrazin 2004 | Not reported | Consecutive | Mentioned (clinical criteria, serum cortisol, UFC, 2 and 8 mg dexamethasone test, ACTH, CT/MRI) | Mentioned (cortisol; 3 weeks postoperatively) | Not reported |
| Mortini 2005 | Not reported | Consecutive | Not mentioned | Mentioned (hypocortisolism requiring glucocorticoid substitution, serum cortisol, UFC, low dose dexamethasone test; normal during minimal 6 months) | Not reported |
| Nakane 1987 | Not reported | Consecutive | Not mentioned | Mentioned (clinical improvement, plasma cortisol) | Not reported |
| Nemergut 2005 | Not reported | Consecutive | Not mentioned | Mentioned | Not reported |
| Norris 1997 | Not reported | Consecutive | Mentioned (CT/MRI, IPSS) | Mentioned (cortisol) | Not reported |
| Patil 2008 | Not reported | Minimum follow-up of 6 months | Mentioned (24-h UFC, MRI, IPSS, high dose dexamethasone test) | Mentioned (24-h UFC, continued need for cortisol replacement; within 72h) | Not reported |
| Patil 2008 (repeat surgery only) | 4 (10.0%) | Minimum of 3 months remission before recurrence | Mentioned (24-h UFC, clinical criteria, histology first surgery, MRI, IPSS, high dose dexamethasone test) | Mentioned (24-h UFC, continued need for glucocorticoid replacement) | Not reported |
| Petruson 1997 | 0 (0%) | Consecutive | Mentioned (UFC, morning serum cortisol, circadian rhythm, low and high dose dexamethasone test, metyrapone test, scintigraphy adrenals, CT/MRI, IPSS) | Mentioned (24-h UFC, circadian rhythm of serum cortisol; 2-5 weeks postoperatively, 48 after cortisol replacement) | Not reported |
| Pieters 1989 | Not reported | Unclear | Mentioned (dexamethasone test, ACTH, plasma cortisol, CT, CRH test) | Mentioned (cortisol; 10-20d postoperatively, after discontinuation of replacement several days) | No co-interventions |
| Pikkarainen 1999 | Not reported | Consecutive | Mentioned (clinical criteria, laboratory, imaging, histology) | Mentioned | 16x radiotherapy post-surgery |
| Pimentel-Filho 2005 | Not reported | Not consecutive | Mentioned (MRI, histology, IHC) | Mentioned (serum cortisol, 24-h UFC, 1 mg dexamethasone test; 12-24 months postoperatively) | Not reported |
| Post 1995 | 2 cured patients in the first month (5.9%) | Consecutive | Mentioned (24-h UFC, low dose dexamethasone test, high dose dexamethasone test, ACTH, CRH test, IPSS, MRI/CT) | Mentioned (24-h UFC; 1-3 weeks postoperatively after at least 36h without cortisol replacement) | Not reported |
| Potts 2014 | 22 (24.2%) | Consecutive | Mentioned (MRI, IPSS, biochemical studies) | Not mentioned | 2x radiosurgery, 1x craniotomy, 3x multiple pituitary treatments, 5x adrenalectomy |
| Powell 2017 | 0 (0.0%) | Consecutive | Mentioned (clinical criteria, ACTH, cortisol, high dose dexamethasone test, biochemistry blood and urine, MRI/CT) | Mentioned (basal 9:00 cortisol level; within 3 months postoperatively) | 9x unilateral adrenalectomy, 3x bilateral adrenalectomy, 2x presurgical treatment with medication |
| Rollin 2007 | 8 (7.4%), only assessed for initial remission rate and clinical data | Consecutive | Mentioned (UFC, diurnal rhythm, low dose dexamethasone test, 1 mg dexamethasone test, midnight cortisol, ACTH, vasopressin test, high dose dexamethasone test, MRI/CT, IPSS) | Mentioned (clinical and laboratory signs of adrenal insufficiency, glucocorticoid dependence, 1 mg dexamethasone test, clinical remission of hypercortisolism; 10-12d postoperatively) | Not reported |
| Salassa 1978 (abstract only) | Not reported | Only patients with radiologically visible adenoma | Not mentioned | Not mentioned | Not reported |
| Semple, C.G. 1984 | 0 (0.0%) | Consecutive | Mentioned (UFC, diurnal rhythm of cortisol and ACTH, six day dexamethasone test, insulin-induced hypoglycemia test, CT) | Mentioned (return to normal physique and resolution of dermatological abnormalities, early-morning plasma cortisol, insulin-induced hypoglycemia test; 1 week postoperatively) | Not reported |
| Semple, P.L. 1999 | Not reported | Consecutive | Mentioned (endocrinological function tests, MRI) | Not mentioned | Not reported |
| Shah 2006 | 8 (11.6%) | Consecutive | Mentioned (low dose dexamethasone test, high dose dexamethasone test, imaging (preferably MRI), IPSS) | Mentioned (basal serum cortisol; 5-7d postoperatively) | Not reported |
| Shimon 2002 | 3 (3.7%) | Consecutive | Mentioned (IPSS, UFC, serum cortisol, low dose dexamethasone test, high dose dexamethasone test, ACTH, MRI) | Mentioned (resolution of presenting features of hypercortisolism, 24-h UFC 48-h low dose dexamethasone treatment; 4-6 weeks postoperatively after 24h without cortisol replacement) | 2x presurgical treatment with medication, 1x bilateral adrenalectomy, 1x radiotherapy, 1x distal pancreatectomy |
| Shirvani 2016 | Not reported | Consecutive | Mentioned (clinical criteria, ACTH, 1 mg dexamethasone test, 24-h UFC, low and high dose dexamethasone test, MRI/CT) | Mentioned (24-h UFC, basal serum cortisol; 1d postoperatively) | Not reported |
| Sonino 1996 | 12 (10.4%) | Minimum follow-up of 24 months | Mentioned (clinical criteria, UFC, circadian rhythm, low dose dexamethasone test, high dose dexamethasone test, metyrapone test, insulin-induced hypoglycemia, CRH test, ACTH, MRI/CT, IPSS) | Mentioned (regression of clinical and biochemical characteristics, 24-h UFC, low dose dexamethasone test; within 5-15 days postoperatively, at least 24h after withdrawal of replacement) | Not reported |
| Sudhakar 2004 | Not reported | Consecutive | Not mentioned | Mentioned (serum cortisol; within a few days postoperatively) | Not reported |
| Swearingen 1999 | 3 (1.9%) | Consecutive | Mentioned (clinical criteria, ACTH, suppression test) | Mentioned (fasting cortisol, 24-h UFC; within 10d postoperatively) | 7x previous radiotherapy or transsphenoidal surgery |
| Valderrabano 2014 (repeat surgery only) | 7 within 3 months (26.9%) | All patients with persistent or recurrent disease | Mentioned (UFC, circadian rhythm, ACTH, 1 mg dexamethasone test, low dose dexamethasone test, imaging sella, IPSS) | Mentioned (serum cortisol, UFC; 8-12d postoperatively, 24 h after discontinuation replacement) | Not reported |
| Vallette-Kasic 2000 | Not reported | Microadenomas only | Mentioned (clinical criteria, ACTH, plasma cortisol UFC, low dose dexamethasone test, high dose dexamethasone test, IPSS, MRI) | Mentioned (clinical cure, 8:00h plasma cortisol, UFC, plasma ACTH, cortisol nyctohemeral variations; day 8 postoperatively, without previous cortisol replacement) | Not reported |
| Witek 2012 | Not reported | Consecutive | Mentioned (clinical criteria, UFC or 17-OHCS, morning serum cortisol, circadian rhythm, ACTH, low dose dexamethasone test, high dose dexamethasone test, MRI, CRH test) | Mentioned (clinical and biochemical evidence of eucortisolemia or adrenal insufficiency: serum cortisol, UFC, circadian rhythm, 1 mg dexamethasone test (end of FU)) | Not reported |
| Witek 2016 | Not reported | Consecutive | Mentioned (clinical criteria, UFC, morning serum cortisol, circadian rhythm, ACTH, 1 mg dexamethasone test, high dose dexamethasone test, MRI, CRH test, IPSS) | Mentioned (clinical and biochemical evidence of adrenal insufficiency or biochemical evidence of eucortisolemia: UFC, morning serum cortisol, ACTH, circadian rhythm, 1 mg dexamethasone test) | Not reported |
| Yap 2002 | 4 (3.6%) | Consecutive | Mentioned (clinical criteria, 24-h UFC, ACTH, midnight cortisol, low and high dose dexamethasone test, CRH test, IPSS, CT/MRI) | Mentioned (plasma cortisol; 3-4d postoperatively, 24h after cortisol replacement) | 52x pre-surgical medical treatment |
| ***Endoscopic surgery*** |  |  |  |  |  |
| Alahmadi 2013 | Not reported | Consecutive | Mentioned (clinical criteria, 1 mg and 8 mg dexamethasone test, 24-h UFC, midnight salivary cortisol, serum cortisol, ACTH, MRI, IPSS) | Mentioned (24-h UFC, 1 mg dexamethasone test, or dependent on cortisol replacement; 3 and 6 months postoperatively) | Not reported |
| Atkinson 2008 | Not reported | Only micro-adenoma, follow-up >3 months | Not mentioned | Mentioned (postoperative hypocortisolism) | No co-interventions |
| Berker 2013 | Not reported | Consecutive | Mentioned (ACTH, fasting cortisol, low dose dexamethasone test, high dose dexamethasone test, IPSS, MRI/CT) | Mentioned (fasting plasma cortisol (day 7 postop), low dose dexamethasone test; 3 and 6 months postoperatively) | No presurgical treatment with medication |
| Cebula 2017 | Not reported | Consecutive | Mentioned (clinical criteria, endocrinological confirmation of hypercortisolism and dynamic test, MRI, IPSS) | Mentioned (serum cortisol, requiring replacement; 2nd postoperative day) | Not reported |
| Cheng 2011 | Not reported | Consecutive | Mentioned (serum cortisol, 24-h UFC, ACTH, MRI) | Mentioned (24-h UFC, morning serum cortisol, ACTH) | Not reported |
| Dehdashti 2007 | Not reported | Consecutive | Mentioned (2 mg dexamethasone test, high dose dexamethasone test, UFC, MRI, IPSS) | Mentioned (plasma cortisol, 1 mg dexamethasone test, 24-h UFC; 1 and 3 months postoperatively) | 4x presurgical treatment with medication |
| Dehdashti 2008 | Not reported | Consecutive | Mentioned (24-h UFC, plasma cortisol, ACTH, MRI/CT) | Mentioned (early morning cortisol, requiring substitution within 48h postoperatively, low dose dexamethasone, UFC; at 4 and 6 week follow-up) | Not reported |
| D’Haens 2009 | Not reported | Consecutive | Mentioned (ACTH, 24-h UFC, circadian rhythm plasma cortisol, 1 mg dexamethasone test, MRI, IPSS) | Mentioned (serum ACTH and cortisol, 24-h UFC; at least 6 weeks postoperatively) | Not reported |
| Frank 2006 | Not reported | Consecutive | Mentioned (fasting cortisol, ACTH, 24-h UFC, MRI/CT) | Mentioned (early morning cortisol, requiring substitution, UFC; day 3, months 1, 3, and 6, unclear on which measurement remission status is based) | Not reported |
| Gondim 2010 | Not reported | Consecutive | Mentioned (ACTH, cortisol, 24-h UFC, MRI/CT) | Mentioned (early morning cortisol, requiring substitution within 48h postoperatively, low dose dexamethasone test, UFC; at 4 and 6 week follow-up) | Not reported |
| Hofstetter 2011 | Not reported | Consecutive | Mentioned (MRI, cortisol, ACTH, 24-h UFC, low dose dexamethasone test) | Mentioned (early morning cortisol, UFC; within 48h postoperatively) | Not reported |
| Hwang 2009 | 2 (10.0%) | Consecutive | Mentioned (clinical criteria, laboratory, MRI, IPSS, ACTH, basal cortisol, UFC, high dose dexamethasone test, CRH test) | Mentioned (free of symptoms of hypercortisolism and dependency on substitution or biochemical eucortisolemia without replacement; 6 months postoperatively) | Not reported |
| Jho 2001 | Not reported | Consecutive | Not mentioned | Not mentioned | Not reported |
| Kabil 2005 | Not reported | Consecutive | Not mentioned | Not mentioned | Not reported |
| Kuo 2015 | Not reported | Only patients with adenoma visible on MRI | Mentioned (24-h UFC, serum cortisol, other tests according to consensus guidelines, MRI) | Mentioned (UFC, serum cortisol, ACTH; average 72h postoperatively) | No co-interventions |
| Leach 2010 | Not reported | Consecutive | Not mentioned | Mentioned (morning serum cortisol; 2-3 weeks postoperatively) | Not reported |
| Mamelak 2012 | 0 (0.0%) | Consecutive | Not mentioned | Mentioned (morning cortisol in absence of exogenous steroid replacement, clinical symptoms of adrenal insufficiency and requirement for replacement therapy; within 72h postoperatively) | Not reported |
| Masopust 2017 | Not reported | Unclear | Not mentioned | Not mentioned | Not reported |
| Paluzzi 2014 | Not reported | Consecutive | Mentioned (serum cortisol, ACTH, 24-h UFC, late night salivary cortisol, low and high dose dexamethasone test, MRI/CT, IPSS) | Mentioned (morning cortisol, requiring cortisol replacement, 24-h UFC, disappearance of symptoms; <1 week and at 6 months) | Not reported |
| Rudnik 2007 (abstract only) | Not reported | Unclear | Not mentioned | Not mentioned | Not reported |
| Sarkar 2016 | 4 (6.3%) | Consecutive | Mentioned (clinical criteria, 24-h UFC, low dose dexamethasone test, circadian rhythm, high dose dexamethasone test, MRI, PET) | Mentioned (hypocortisolemia with requirement of replacement or eucortisolemia, 1 mg dexamethasone test; 3 months postoperatively, 72h discontinued replacement) | 30x presurgical treatment with medication |
| Senior 2008 | Not reported | Consecutive | Not mentioned | Mentioned | Not reported |
| Shin 2015 | 6 (10.7%) | Consecutive | Mentioned (1 mg dexamethasone test, ACTH, CRH test, 24-h UFC, late night salivary cortisol, IPSS, MRI) | Mentioned (symptoms of adrenal insufficiency requiring replacement, fasting cortisol; within 36h postoperatively or at 8:00 within 2 weeks postoperatively) | Not reported |
| Starke 2013 | Not reported | Consecutive | Mentioned (24-h UFC, serum cortisol, MRI, IPSS) | Mentioned (24-h UFC, early morning cortisol, without replacement; 1d postoperatively) | Not reported |
| Torales 2014 | Not reported | Minimum follow-up of 12 months, postoperative mortality also included | Mentioned (ACTH, cortisol, MRI, IPSS) | Mentioned (basal plasma cortisol, UFC, midnight salivary cortisol, 1 mg dexamethasone test; 3 months postoperatively) | Not reported |
| Wagenmakers 2013 | Not reported | Consecutive | Mentioned (clinical criteria, 24-h UFC, diurnal rhythm plasma or salivary cortisol, 1 mg dexamethasone test, ACTH, MRI, IPSS, CRH test, high dose dexamethasone test) | Mentioned (disappearance of clinical symptoms of hypercortisolism, basal plasma cortisol after glucocorticoid withdrawal of 24-48h, 1 mg dexamethasone test; within 3 months postoperatively) | 78x presurgical treatment with medication |

ACTH=adrenocorticotropic hormone, CRH=corticotropin-releasing hormone, CT=computed tomography, IHC=immunohistochemistry, IPSS=inferior petrosal sinus sampling, MRI=magnetic resonance imaging, OHCS=hydroxycorticosteroids, OHS=hydroxysteroids, UFC=urinary free cortisol
