# Supplementary material for: Endoscopic vs. microscopic transsphenoidal surgery for Cushing’s disease: a systematic review and meta-analysis
Source: Pituitary. 2018 May 16;21(5):524–34. doi: 10.1007/s11102-018-0893-3 (PMC6132967; doi:10.1007/s11102-018-0893-3)
Supplement: Supplementary file 4 — Supplementary material 4 (DOCX 43 KB) [file 11102_2018_893_MOESM4_ESM.docx]

**Pituitary
Endoscopic vs. microscopic transsphenoidal surgery for Cushing’s disease: a systematic review and meta-analysis.**

Leonie H.A. Broersen^1,2^, Nienke R. Biermasz^1,2^, Wouter R. van Furth^2,3^, Friso de Vries^1,2^, Marco J.T. Verstegen^2,3^, Olaf M. Dekkers^1,4^, Alberto M. Pereira^1,2^

^1^Department of Medicine, division of Endocrinology, Leiden University Medical Centre, Albinusdreef 2, 2333 ZA, Leiden, The Netherlands
^2^Center for Endocrine Tumors Leiden (CETL), Leiden University Medical Center, Albinusdreef 2, 2333 ZA, Leiden, The Netherlands
^3^Department of Neurosurgery, Leiden University Medical Centre, Leiden, Albinusdreef 2, 2333 ZA, The Netherlands
^4^Department of Clinical Epidemiology, Leiden University Medical Center, Leiden, Albinusdreef 2, 2333 ZA, The Netherlands

Corresponding author: L.H.A. Broersen, [L.H.A.Broersen@lumc.nl](mailto:L.H.A.Broersen@lumc.nl), +31 (0)71-5263082

Online Resource 4: study outcomes.

|  | **Number of patients total (Number of patients with repeat surgery)** | **Remission  (Remission after repeat surgery)** | **Recurrence** | **Mortality <3 months /Mortality >3 months** | **Complications^a^** | **Number of microadenomas** | **Remission /Recurrence for microadenomas** | **Number of macroadenomas** | **Remission /Recurrence for macroadenomas** |
| --- | --- | --- | --- | --- | --- | --- | --- | --- | --- |
| ***Microscopic surgery*** |  |  |  |  |  |  |  |  |  |
| Acebes 2007 | 44 | 39 | 3 | nr | H:37 | 27 | nr | 10 | nr |
| Alahmadi 2013 | 25 | 16 | nr | nr | C:0, A:3, D:7 | 17 | 10/nr | 8 | 6/nr |
| Alexandraki 2013 | 124 | 84 | 20 | 1/3 | nr | 103 | 75/17 | 21 | 9/3 |
| Alwani 2010 | 79 | 51 | 10 | 0/8 | A:22, PD:2 | 58 | 42/8 | 21 | 9/2 |
| Ammini 2011 | 81 (9) | 54 (6) | 10 | 2^b^ | nr | 68 | 46/nr | 13 | 8/nr |
| Arnott 1990 | 28 (2) | 24 (2) | 3 | nr | C:5, M:1, A:4, H:6, PD:3 | nr | nr | nr | nr |
| Asuzu 2017 | DP (23) | (21) | - | - | - | - | - | - | - |
| Atkinson 2008 | 21 | 18 | 2 | nr | C:2, H:18, TD:3 | 21 | 18/2 | 0 | - |
| Bakiri 1996 | 50 (2) | 36 (1) | 3 | 2/2 | M:2, A:6, H:3, TD:19, PD:3 | nr | nr | nr | nr |
| Barbetta 2001 | 68 (6) | 61 (1) | 13 | nr | nr | 63 | nr | 5 | nr |
| Barbot 2013 | 57 | 39 | 15 | nr | nr | nr | nr | nr | nr |
| Barzaghi 2007 | 288 | nr | nr | 1/nr | M:0, T:4 | nr | nr | nr | nr |
| Bay 1988 | 36 | 31 | 3 | 0/nr | C:6, M:2, A:5, H:22, B:1, TD:15, PD:1, P:1 | 27 | nr/3 | 1 | nr/0 |
| Bigos 1980 | DP 24 | - | - | - | B:4, TD:1 | - | - | - | - |
| Blevins 1998 | 96 (18) | 82 (4) | 13 | 0/nr | M:0 | 75 | 68/8 | 21 | 14/5 |
| Burkhardt 2013 | 42 | 40 | nr | nr | nr | 27 | nr | 4 | nr |
| Chandler 2016 | 275 (33) | 219 (15) | 37 | 0/3 | C:1, M:1, H:190, TD:14, PD:2 | 137 | 122/nr | 35 | 23/nr |
| Chee 2001 | 61 (13) | 48 (5) | 7 | 0/3 | C:8, M:0, A:12, H:9, B:1, TD:13 | nr | nr | nr | nr |
| Chen 2003 | 174 (5) | 142 (1) | 22 | 0/4 | C:0, M:0, A:0 | 133 | 132/13 | 29 | 10/9 |
| Cheng 2011 | 18 | 9 | nr | 0/nr | nr | 8 | 6/nr | 10 | 3/nr |
| Ciric 2012 | 121 | 101 | 9 | 0/nr | C:6, M:1, S:2, H:101, T:4, B:5, TD:17 | 108 | 97/9 | 13 | 4/0 |
| D’Haens 2009 | 13 (5) | 6 (2) | nr | 0/nr | PD:0 | nr | nr | nr | nr |
| Donofrio 2016 | 142 | 114 | nr | nr | C:1, M:0, T:1, B:3, D:15, P:1 | nr | nr | nr | nr |
| Erem 2003 | 25 (1) | 21 (1) | 3 | nr | nr | nr | nr | nr | nr |
| Esposito 2006 | 40 (2) | 31 (2) | 1 | 1/nr | C:1, B:1, TD:4, PD:1 | 31 | 25/nr | 9 | 6/nr |
| Flitsch 2003 | 147 (7) | 137 (7) | 8 | 0/nr | C:3, A:19, B:2, PD:5 | nr | nr | nr | nr |
| Gazioglu 2008 | 26 (2) | 21 (2) | nr | 0/nr | A:0 | nr | nr | nr | nr |
| Gsponer 1999 | 15 | 13 | 1 | nr | A:1, H:13, D:1 | nr | nr | nr | nr |
| Guilhaume 1988 | 64 | 42 | 6 | 1/nr | C:5, M:1, A:0, B:4, TD:31 | nr | nr | nr | nr |
| Hammer 2004 | 227 | 189 | nr | nr | nr | nr | nr | nr | nr |
| Hardy 1979 | 25 | 19 | 2 | nr | H:3 | nr | nr | nr | nr |
| Hofmann 2008 | 426 (90) | 280 (24) | 42 | 3/nr | C:2, M:4, A:4, T:2, D:4 | 330 | 257/39 | 39 | 23/3 |
| Honegger 2012 | 83 (9) | 70 (7) | 5 | 1/nr | A:11, T:2, TD:11, PD:2 | 72 | 63/5 | 11 | 7/0 |
| Hoybye 2004 | 34 | 31 | 2 | 0/1 | M:0, A:5, H:17, T:1, B:0, TD:1, PD:1 | nr | nr | nr | nr |
| Huan 2014 | 84 | 59 | 17 | 0/nr | TD:44 | 25 | 24/nr | 59 | 35/nr |
| Imaki 2001 | 49 | 47 | 7 | 0/2 | nr | 29 | 28/4 | 12 | 12/2 |
| Inder 2003 | 15 (1) | 13 (0) | 0 | 0/nr | H:2, TD:8 | nr | nr | nr | nr |
| Jagannathan 2009 | 483 | 453 | 6 | 0/1 | C:4, S:30, M:1, B:1, TD:15, PD:1 | nr | nr | nr | nr |
| Jehle 2008 | 193 | 156 | 21 | 0/10 | C:4, S:8, T:1, B:2, TD:35 | nr | nr | nr | nr |
| Knappe 2011 | 16 (2) | 16 (2) | 0 | 0/0 | A:1, H:13, T:1, B:1, TD:1 | 17 | 17/0 | 1 | 1/0 |
| Kristof 2002 | 27 | 22 | 2 | 0/nr | S:5, TD:10, PD:2 | 21 | 19/2 | 6 | 3/0 |
| Kurosaki 2000 | 51 (2) | 48 (2) | 2 | nr | C:4, A:2, PD:0 | nr | nr | nr | nr |
| Lampropoulos 2013 | 23 | 16 | 2 | nr | A:0 | 14 | 10/nr | 9 | 6/nr |
| Lüdecke 1985 | 88 (5) | 80 (5) | 5 | 2/2 | nr | 72 | 68/nr | 16 | 12/nr |
| Lüdecke 1991 | 103 | nr | nr | 0/1 | nr | nr | nr | nr | nr |
| Mampalam 1988 | DP | - | - | - | - | 170 | 144/5 | 39 | 25/4 |
| Mehrazin 2004 | 11 | 10 | 1 | 1/nr | C:3, M:2, TD:6, PD:1 | 7 | nr/1 | 4 | nr/0 |
| Mortini 2005 | DP 262 | 203 | 12 | - | - | 213 | 168/nr | 48 | 35/nr |
| Nakane 1987 | 98 | 86 | 8 | 3^b^ | nr | 76 | 73/7 | 17 | 13/1 |
| Nemergut 2005 | DP 180 | - | - | - | TD:40, PD:1 | - | - | - | - |
| Norris 1997 | 100 | 80 | 0 | 0/nr | C:8, M:3 | nr | nr | nr | nr |
| Patil 2008 | 215 | 184 | 32 | nr | nr | nr | nr | nr | nr |
| Patil 2008 (repeat surgery only) | (36) | (22) | - | - | - | - | - | - | - |
| Petruson 1997 | 31 (4) | 24 (1) | 1 | 0/1 | C:0, M:0, A:2, H:3, D:2 | nr | nr | nr | nr |
| Pieters 1989 | 27 | 16 | 4 | nr | nr | nr | nr | nr | nr |
| Pikkarainen 1999 | 43 | 36 | 15 | 3^b^/3 | nr | nr | nr | nr | nr |
| Pimentel-Filho 2005 | 17 | 8 | nr | nr | nr | 13 | 7/nr | 4 | 1/nr |
| Post 1995 | 34 (11) | 19 (4) | 5 | 1/0 | C:1, M:1, A:5, H:1, TD:4, PD:1 | 26 | 17/nr | 4 | 2/nr |
| Potts 2014 | 91 | 53 | nr | nr | C:1, A:1, T:2, D:4 | nr | nr | nr | nr |
| Powell 2017 | 32 (5) | 29 (3) | 4 | 1/1 | nr | nr | nr | nr | nr |
| Rollin 2007 | 103 (14) | 88 (4) | 4 | nr | nr | nr | nr | nr | nr |
| Salassa 1978 (abstract only) | 18 | 16 | nr | nr | nr | nr | nr | nr | nr |
| Semple, C.G. 1984 | 10 | 10 | 1 | nr | C:6, M:0, A:8, H:3, T:1, TD:3, PD:1, P:4 | 9 | 9/1 | 1 | 1/0 |
| Semple, P.L. 1999 | DP 105 | - | - | 1/nr | C:1, S:1, T:4, B:1 | - | - | - | - |
| Shah 2006 | 65 (4) | 40 (0) | 5 | 5/nr | C:4, M:4, T:1 | 30 | 23/nr | 14 | 5/nr |
| Shimon 2002 | 74 (13) | 58 (8) | 3 | nr | C:5, S:1, A:2, H:7, PD:5 | 42 | 33/nr | 3 | 1/nr |
| Shirvani 2016 | 96 (17) | 91 (12) | 21 | 0/nr | C:8, M:1, TD:15, PD:2 | 78 | nr/5 | 18 | nr/1 |
| Sonino 1996 | 103 | 79 | 20 | nr | nr | nr | nr | nr | nr |
| Sudhakar 2004 | 22 (3) | 16 (3) | nr | 0/nr | C:3, M:2, A:9, T:2, B:1, TD:11 | 18 | 16/nr | 4 | 0/nr |
| Swearingen 1999 | 154 (35) | 134 (16) | 10 | 0/6 | C:5, M:3, A:26, H:36, B:1, PD:7 | 137 | 123/7 | 17 | 11/3 |
| Valderrabano 2014 (repeat surgery only) | (26) | (12) | - | - | - | - | - | - | - |
| Vallette-Kasic 2000 | 53 | 43 | 5 | nr | TD:9, PD:0 | nr | nr | nr | nr |
| Witek 2012 | 28 (8) | 22 (1) | nr | nr | A:5, PD:3 | 22 | 17/nr | 6 | 3/nr |
| Witek 2016 | 40 | 32 | nr | nr | nr | nr | nr | nr | nr |
| Yap 2002 | 97 | 61 | 7 | 1/nr | C:8, M:2, T:2, B:6, TD:33, PD:8 | 76 | nr | 10 | nr |
| ***Endoscopic surgery*** |  |  |  |  |  |  |  |  |  |
| Alahmadi 2013 | 17 | 10 | nr | nr | C:2, A:1, D:4 | 10 | 6/nr | 7 | 4/nr |
| Atkinson 2008 | 21 | 18 | 2 | nr | C:3, H:18, TD:3 | 21 | 18/2 | 0 | - |
| Berker 2013 | 69 (25) | 66 (19) | 4 | 0/nr | C:8, M:1, A:2, H:7, TD:7, PD:1 | 43 | 40/4 | 26 | 26/0 |
| Cebula 2017 | 230 | 182 | 18 | nr | C:29, M:0, S:8, A:7, T:4, B:6, TD:51, PD:15 | 106 | 97/9 | 54 | 35/2 |
| Cheng 2011 | 22 | 15 | nr | 0/nr | nr | 10 | 8/nr | 12 | 7/nr |
| Dehdashti 2007 | DP 25 (3) | (3) | 0 | - | C:1, M:0, A:3, H:12, B:0, TD:1 | - | - | - | - |
| Dehdashti 2008 | 27 | 22 | nr | 0/nr | nr | 16 | 16/nr | 8 | 5/nr |
| D’Haens 2009 | 16 (5) | 9 (2) | nr | 0/nr | PD:0 | nr | nr | nr | nr |
| Frank 2006 | 56 | 38 | nr | nr | nr | 31 | 21/nr | 25 | 17/nr |
| Gondim 2010 | 28 (3) | 20 (0) | nr | nr | nr | 20 | 15/nr | 8 | 5/nr |
| Hofstetter 2011 | 18 | 11 | nr | nr | A:4 | 11 | 6/nr | 7 | 5/nr |
| Hwang 2009 | 20 | 16 | 1 | nr | nr | 15 | 11/nr | 5 | 5/nr |
| Jho 2001 | 16 | 11 | nr | nr | nr | nr | nr | nr | nr |
| Kabil 2005 | 28 | 24 | nr | nr | nr | nr | nr | nr | nr |
| Kuo 2015 | 40 | 32 | 3 | nr | C:2, D:0 | nr | nr | nr | nr |
| Leach 2010 | 10 (3) | 7 (1) | nr | nr | nr | nr | nr | nr | nr |
| Mamelak 2012 | 12 | 10 | 0 | nr | nr | nr | nr | nr | nr |
| Masopust 2017 | 41 | 36 | nr | nr | nr | nr | nr | nr | nr |
| Paluzzi 2014 | DP | - | - | - | - | 36 | 30/1 | 21 | 17/0 |
| Rudnik 2007 (abstract only) | 13 | 11 | nr | 0^b^ | nr | nr | nr | nr | nr |
| Sarkar 2016 | 64 (4) | 47 (0) | 4 | 1/nr | C:34, M:2, A:11, H:16, T:1, B:3, TD:8, PD:2 | 45 | 39/nr | 11 | 6/nr |
| Senior 2008 | 20 | nr | nr | nr | C:1, D:3 | nr | nr | nr | nr |
| Shin 2015 | 50 (3) | 40 (2) | 9 | 2/nr | C:2, A:8, T:1, TD:4 | nr | nr | nr | nr |
| Starke 2013 | 61 (5) | 58 (1) | 5 | nr | C:21, A:8, B:1, TD:10, PD:3 | 30 | 29/nr | 15 | 13/nr |
| Torales 2014 | 19 | 16 | nr | nr | A:2 | nr | nr | nr | nr |
| Wagenmakers 2013 | 86 (16) | 62 (7) | 10 | 1^b^ | C:4, S:10, A:22, H:12, T:1, B:11, TD:4 | 55 | 41/nr | 31 | 21/nr |

nr = not reported, DP = double population, only data included in analyses reported. ^a^Complications are coded as follows: C: CSF leak, M: meningitis, S: SIADH, A: anterior pituitary deficiency, H: hydrocortisone dependency, T: thromboembolism, B: bleeding, TD: transient diabetes insipidus, PD: permanent diabetes insipidus, D: diabetes insipidus (unknown duration), P: psychopathology.
^b^Time since surgery not reported.
