# Supplementary material for: Endoscopic vs. microscopic transsphenoidal surgery for Cushing’s disease: a systematic review and meta-analysis
Source: Pituitary. 2018 May 16;21(5):524–34. doi: 10.1007/s11102-018-0893-3 (PMC6132967; doi:10.1007/s11102-018-0893-3)
Supplement: Supplementary file 5 — Supplementary material 5 (DOCX 26 KB) [file 11102_2018_893_MOESM5_ESM.docx]

**Pituitary
Endoscopic vs. microscopic transsphenoidal surgery for Cushing’s disease: a systematic review and meta-analysis.**

Leonie H.A. Broersen^1,2^, Nienke R. Biermasz^1,2^, Wouter R. van Furth^2,3^, Friso de Vries^1,2^, Marco J.T. Verstegen^2,3^, Olaf M. Dekkers^1,4^, Alberto M. Pereira^1,2^

^1^Department of Medicine, division of Endocrinology, Leiden University Medical Centre, Albinusdreef 2, 2333 ZA, Leiden, The Netherlands
^2^Center for Endocrine Tumors Leiden (CETL), Leiden University Medical Center, Albinusdreef 2, 2333 ZA, Leiden, The Netherlands
^3^Department of Neurosurgery, Leiden University Medical Centre, Leiden, Albinusdreef 2, 2333 ZA, The Netherlands
^4^Department of Clinical Epidemiology, Leiden University Medical Center, Leiden, Albinusdreef 2, 2333 ZA, The Netherlands

Corresponding author: L.H.A. Broersen, [L.H.A.Broersen@lumc.nl](mailto:L.H.A.Broersen@lumc.nl), +31 (0)71-5263082

Online Resource 5: results of meta-analyses comparing microscopic and endoscopic surgery – sensitivity analyses.

|  | **Excluding inadequate selection of patients and >5% loss to follow-up** | | **Only including study period starting from ≥2000** | | **Only including studies with specific criteria for diagnosis of Cushing’s disease** | | **Only including studies with low dose dexamethasone test for remission** | | **Only including studies testing remission three to six months postoperatively** | |
| --- | --- | --- | --- | --- | --- | --- | --- | --- | --- | --- |
|  | **M % (95% CI); N** | **E % (95% CI); N** | **M % (95% CI); N** | **E % (95% CI); N** | **M % (95% CI); N** | **E % (95% CI); N** | **M % (95% CI); N** | **E % (95% CI); N** | **M % (95% CI); N** | **E % (95% CI); N** |
| ***Meta-analysis of surgical outcomes*** |  | |  | |  | |  | |  | |
| Remission | 82.0 (78.6-85.2); 39 | 77.3 (69.8-84.0); 15 | 74.5 (67.5-81.0); 13 | 80.2 (73.6-86.1); 15 | 79.7 (75.8-83.4); 44 | 77.0 (69.2-84.0); 14 | 78.0 (73.8-81.9); 23 | 78.7 (67.5-88.1); 7 | 76.3 (69.5-82.5); 9 | 78.7 (64.7-90.1); 6 |
| Hydrocortisone dependency | 44.4 (27.0-62.6); 14 | 15.9 (10.8-21.8); 3 | - | 20.8 (14.6-27.6); 3 | 37.2 (17.8-58.9); 14 | 18.2 (13.5-23.4); 4 | 14.7 (10.2-19.9); 4 | 18.2 (13.5-23.4); 4 | - | 16.7 (10.7-23.6); 2 |
| Recurrence | 9.2 (6.1-12.7); 30 | 8.8 (5.6-12.5); 5 | 23.0 (14.7-32.5); 5 | 9.0 (5.8-12.7); 7 | 12.4 (9.1-16.0); 37 | 10.6 (6.8-15.1); 7 | 12.6 (9.2-16.4); 18 | 9.9 (5.7-14.9); 3 | 12.8 (7.6-18.9); 8 | 6.5 (2.5-11.9); 3 |
| Short term mortality | 0.0 (0.0-0.0); 25 | 0.0 (0.0-1.2); 4 | 0.0 (0.0-1.7); 4 | 0.4 (0.0-2.2); 6 | 0.0 (0.0-0.0); 26 | 0.4 (0.0-2.2); 6 | 0.1 (0.0-0.6); 13 | 0.2 (0.0-2.2); 3 | 0.3 (0.0-1.1); 5 | 0.5 (0.0-2.8); 2 |
| Remission after repeat surgery | 57.3 (44.4-69.8); 15 | 47.9 (22.0-74.2); 7 | 67.4 (29.2-96.9); 5 | 48.9 (18.7-79.4); 7 | 51.6 (36.8-66.3); 22 | 43.3 (17.2-71.0); 8 | 47.6 (27.7-67.9); 10 | 44.2 (9.0-82.4); 5 | 28.9 (20.6-37.9); 3 | 65.7 (45.6-83.6); 2 |
| ***Meta-analysis of complications*** |  | |  |  |  | |  | |  |  |
| Cerebrospinal fluid leak | 5.0 (2.6-8.0); 21 | 11.3 (5.2-19.0); 7 | 0.4 (0.0-2.0); 3 | 14.1 (5.7-25.0); 9 | 4.5 (2.0-7.7); 19 | 13.8 (3.9-27.7); 8 | 2.0 (0.1-5.8); 6 | 14.7 (1.6-35.9); 5 | 0.4 (0.0-1.3); 3 | 27.1 (20.1-34.7); 3 |
| Meningitis | 0.4 (0.0-1.3); 18 | 0.0 (0.0-0.6); 3 | - | 0.1 (0.0-1.0); 4 | 0.3 (0.0-1.1); 17 | 1.5 (0.0-4.5); 3 | 0.3 (0.0-1.4); 5 | 1.5 (0.0-4.5); 3 | 0.6 (0.0-1.5); 3 | 2.2 (0.2-5.7); 2 |
| Syndrome of inappropriate antidiuretic hormone secretion | 3.5 (1.3-6.6); 6 | 5.2 (2.9-8.0); 2 | - | - | 4.2 (1.7-7.6); 5 | - | 3.8 (1.7-6.5); 3 | - | - | - |
| Anterior pituitary hormone deficiency | 12.4 (6.7-19.2); 16 | 10.3 (3.4-19.8); 7 | 3.6 (0.0-11.7); 5 | 9.8 (4.7-16.1); 9 | 8.7 (3.7-15.2); 19 | 13.6 (7.7-20.6); 8 | 7.4 (2.3-14.6); 12 | 11.9 (4.6-21.5); 6 | 1.4 (0.5-2.7); 4 | 8.0 (4.0-13.0); 4 |
| Thromboembolism | 1.4 (0.4-2.7); 11 | 1.5 (0.3-3.3); 2 | 1.2 (0.1-3.2); 2 | 1.5 (0.3-3.3); 3 | 0.7 (0.0-2.1); 8 | 1.4 (0.1-3.9); 3 | 0.4 (0.0-1.2); 4 | 1.3 (0.0-4.1); 2 | 0.5 (0.0-1.5); 2 | - |
| Bleeding | 2.0 (0.7-3.8); 15 | 3.4 (1.7-5.6); 4 | - | 2.1 (0.7-4.1); 4 | 2.5 (0.8-4.9); 12 | 5.2 (2.5-8.6); 4 | 0.7 (0.0-2.3); 3 | 6.8 (3.3-11.3); 3 | - | - |
| Transient diabetes insipidus | 19.3 (12.5-27.1); 19 | 11.3 (4.8-19.9); 5 | - | 12.9 (7.9-18.9); 6 | 22.8 (14.5-32.2); 20 | 9.1 (5.7-13.2); 6 | 19.9 (11.6-29.7); 6 | 7.7 (4.5-11.6); 4 | 28.4 (21.8-35.4); 3 | 11.3 (6.3-17.3); 2 |
| Permanent diabetes insipidus | 2.7 (1.0-5.0); 17 | 4.2 (2.2-6.7); 4 | - | 4.0 (2.2-6.3); 5 | 3.3 (1.2-6.2); 17 | 2.3 (0.4-5.2); 4 | 4.3 (2.3-6.8); 7 | 2.2 (0.2-5.7); 2 | 3.1 (0.3-7.6); 2 | 2.2 (0.2-5.7); 2 |
| Psychopathology | 0.7 (0.0-3.1); 3 | - | - | - | 0.7 (0.0-3.1); 3 | - | - | - | - | - |
| ***Meta-analysis of surgical outcomes according to tumor size*** | | |  | |  | |  | |  | |
| Remission for microadenoma | 85.8 (81.3-89.9); 18 | 83.8 (74.9-91.3); 11 | 76.0 (62.8-87.3); 6 | 88.4 (80.5-94.6); 9 | 84.2 (77.7-89.9); 19 | 82.4 (74.0-89.7); 12 | 82.9 (76.2-88.8); 15 | 85.1 (73.5-94.1); 6 | 82.6 (76.3-88.1); 6 | 81.1 (70.5-89.9); 6 |
| Recurrence for microadenoma | 5.6 (3.4-8.2); 8 | 8.0 (4.2-12.9); 3 | - | 8.0 (4.2-12.9); 3 | 10.6 (6.3-15.7); 10 | 6.8 (1.7-14.4); 2 | 11.8 (7.1-17.4); 8 | - | 12.7 (9.5-16.3); 4 | 6.8 (1.7-14.4); 2 |
| Remission for macroadenoma | 64.2 (55.4-72.7); 17 | 76.0 (63.3-87.0); 10 | 61.6 (50.8-71.9); 5 | 76.9 (59.8-90.8); 8 | 58.5 (48.0-68.7); 18 | 77.6 (64.2-89.0); 11 | 60.6 (49.5-71.3); 15 | 72.8 (47.4-92.7); 6 | 66.9 (58.1-75.2); 6 | 81.9 (59.6-97.3); 6 |
| Recurrence for macroadenoma | 4.6 (0.0-15.1); 8 | 1.5 (0.0-6.4); 3 | - | 1.5 (0.0-6.4); 3 | 18.2 (2.9-39.2); 10 | 0.0 (0.0-4.4); 2 | 21.7 (5.7-42.4); 8 | - | 12.8 (3.1-25.9); 4 | 0.0 (0.0-4.4); 2 |

M = microscopic surgery, E = endoscopic surgery, CI = confidence interval, N = number of studies per analysis
